# Ligand-dependent enhancer activation indirectly modulates non-target promoters in a chromatin domain

**DOI:** 10.7554/eLife.102417

**Published:** 2026-07-07

**Authors:** Darshika Bohra, Zubairul Islam, Sundarraj Nidharshan, Aprotim Mazumder, Dimple Notani

**Affiliations:** 1 Tata Institute of Fundamental Research Hyderabad Hyderabad India; 2 National Centre for Biological Sciences, Tata Institute for Fundamental Research Bangalore India; 3 Sastra Deemed University Thanjavur India; 4 School of Biotechnology, Amrita Vishwa Vidyapeetham Kollam India; University of Pennsylvania United States; National Cancer Institute United States

**Keywords:** breast cancer, estrogen, MCF-7, gene regulation, steroid hormone signaling, transcription, enhancers, Human, MCF-7 cell line

## Abstract

Transcription activation of genes by estrogen is driven by enhancers, which are often located within the same topologically associating domain (TAD) as non-targeted promoters. We investigated how acute enhancer-driven activation affects neighbouring non-target genes within the same TAD. Using single-molecule RNA FISH (smFISH), we tracked the transcription of TFF1 (enhancer-target gene) and TFF3 (non-target gene) during estrogen stimulation. We observed mutually exclusive expression patterns: TFF1 expression peaked at 1 hr, while TFF3 reached its peak at 3 hr after TFF1 activation had diminished. Chromatin looping data indicated that the enhancer loops with the TFF1 gene but not TFF3, suggesting that TFF3 upregulation is not due to direct enhancer-promoter interactions. CRISPR deletion of the enhancer affected TFF1 transcription more acutely than TFF3. 1,6-hexanediol (HD) exposure suggested that the TFF1 enhancer:promoter undergoes a potential ERα-mediated condensate formation, which sequesters the transcriptional machinery and inhibits TFF3 expression. As estrogen signaling fades at 3 hr, TFF1 expression declines while TFF3 expression increases. Our findings reveal that enhancer-driven activation can indirectly repress neighboring genes within the same TAD, highlighting a dynamic shift in gene expression as signaling progresses.

## Introduction

Acute transcriptional activation of ligand-induced genes drives downstream signaling responses. Similar to development-specific genes, signaling-induced genes are also driven by enhancers (Hah et al., 2013; Li et al., 2013; Liu et al., 2014; Uyehara and Apostolou, 2023). Upon binding with ligand-induced transcription factors (TFs), these enhancers loop with their target promoters mostly in the same TAD (Buecker et al., 2014; Bulger and Groudine, 1999; Chepelev et al., 2012; Furlong and Levine, 2018; Oh et al., 2021; Panigrahi and O’Malley, 2021; Ptashne, 1986; Sanyal et al., 2012; Yan et al., 2018). Enhancer: promoter pairing is thought to be specific and forms the basis of noise-free gene activation of a subset of genes crucial for signaling response (Bojcsuk et al., 2017; Chen et al., 2018; Friedman et al., 2024; Galouzis and Furlong, 2022; Zabidi et al., 2015). Due to such specificity, gene transcription occurs in waves of early and late responsive genes (Fowler et al., 2011; Yamamoto and Alberts, 1976). Often, the protein factors translated from early genes regulate the expression of late responsive genes (Dixon et al., 1996; Freter et al., 1996; Herschman, 1991; Williams et al., 1999; Winkles, 1997; Winston and Pledger, 1993). However, it is not known if the genes that are activated early in the signaling time course are spatially related to late activating genes. Furthermore, if the spatial proximity of any gene to an early gene is enough to cause the temporal differential transcription due to sequestration of the transcriptional machinery from late gene to early gene is poorly understood. TFs, after binding to cognate DNA motifs, recruit RNA polymerase machinery and co-activators for gene activation (Levine and Tjian, 2003; Liu et al., 2014). This machinery is limited in supply and may become sequestered from other genomic regions (Koşar and Erbaş, 2022). Furthermore, acute activation of genes is linked with phase-separation of TFs, polymerases, mediators and other co-factors/activators, most of which harbor low complexity regions to promote weak protein-protein interactions driving the formation of TF-condensates (Boehning et al., 2018; Boija et al., 2018; Cai et al., 2019; Chen et al., 2023; Cho et al., 2018; Chong et al., 2018; Chong et al., 2022; Mann and Notani, 2023; Sabari et al., 2018; Shrinivas et al., 2019; Stortz et al., 2020; Stortz et al., 2024). Though the precise stoichiometry of protein molecules in these condensates is unknown, such structures involve multiple molecules of each transcriptional protein. The formation of such phase-separated compartments can potentially act as a sink for transcriptional machinery depriving neighboring promoters and enhancers that are not part of the compartment. Such sequestration would cause indirect suppression of these spatially proximal genes in the same TAD but not the part of the same condensate.

In order to investigate the effect of an enhancer: promoter pair on a neighboring gene within the same TAD, we looked at the paradigmatic model of estrogen signaling in mammary epithelial cells, namely MCF7 (Levenson and Jordan, 1997; Masiakowski et al., 1982). E2-signaling can be induced within minutes by treating the cells with estradiol (E2), which causes activation and repression of several genes across the genome. The peak of E2-mediated signaling occurs 40 min post-induction and starts to decay by 160 min (Hah et al., 2011). This occurs via the binding of estrogen receptor-alpha (ERα) on the enhancers (Li et al., 2013). We selected E2-induced E-P pair TFF1 and its neighboring gene TFF3 on Chr21 as discussed below (Chinery et al., 1996). Several studies have used single-cell methods to interrogate E2-responsive genes, such as GREB1, MYC, and TFF1 as a paradigm to understand their transcriptional response upon estrogen stimulation (Patange et al., 2022; Rodriguez et al., 2019; Stossi et al., 2020). These studies have implicated the cellular states as determinants of transcriptional response and that the long repressive states of TFF1 give rise to expression variability in isogenic lines (Rodriguez et al., 2019).

**Figure 1.**
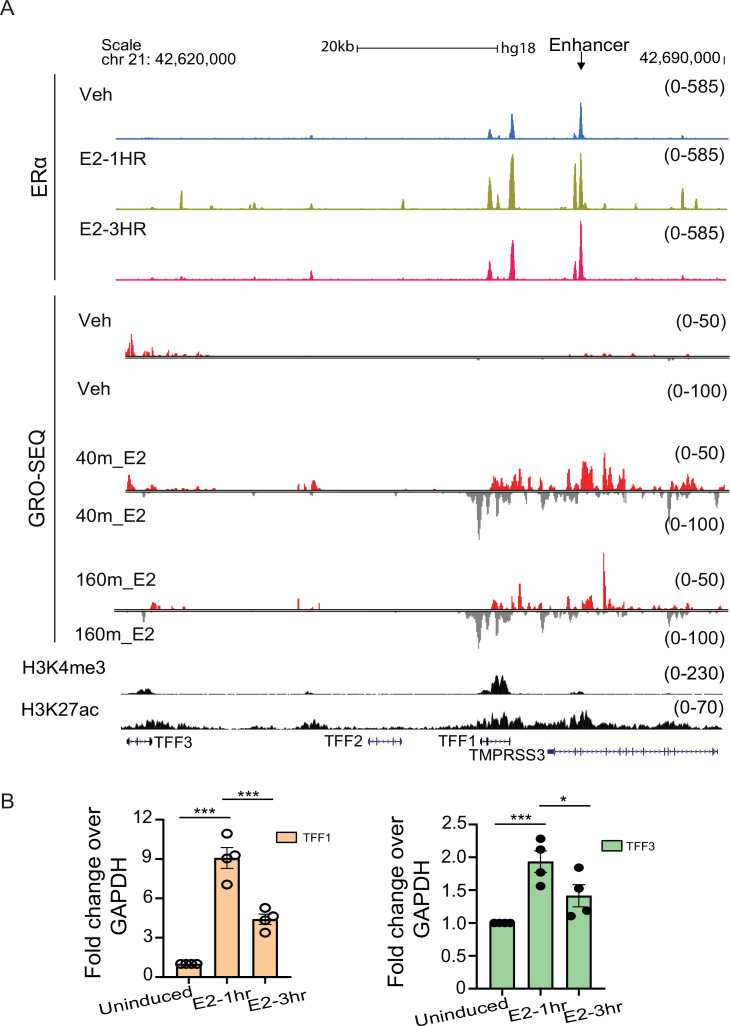
Estrogen receptor-alpha (ERα) binding and ligand-induced gene expression of TFF1 and TFF3 change over the course of estrogen signaling. (**A**) Schematic depicting TFF1 locus, UCSC genome browser snapshots showing the binding of ERα, H3K27ac status, H3K4me3 signal, and GRO-seq signal for robustly estradiol (E2)-induced TFF1 locus. First, second, and third ERα ChIP-seq and GRO-seq tracks are from vehicle-treated, E2-1 hr and E2-3 hr in WT cells, respectively. (**B**) qRT-PCR showing the changes in expression of TFF1 and TFF3 genes during the E2 signaling time course. Error bars denote SEM from four biological replicates. Each dot represents a replicate. *P*-values were calculated by Student’s two-tailed unpaired t-test, and the significance is represented as: *** denotes *p*<0.001, ** denotes *p*<0.01, * denotes *p*<0.05, ns denotes *p*>0.05. Figure 1—source data 1.qRT-PCR values for Figure 1B.

TFF1 is found in a topologically associated domain (TAD) along with a few other genes, including TFF2, TFF3, TMPRSS3, and UBASH3A (Oh et al., 2021; Quintin et al., 2014; Rao et al., 2014). TFF1 and TFF3 are located at 10 kb and 60 kb from enhancer, respectively (Figure 1A), and this locus has been useful in answering the questions about promoter-enhancer interactions and multi-gene regulation (Oh et al., 2021; Quintin et al., 2014).

**Figure 1—figure supplement 1.**
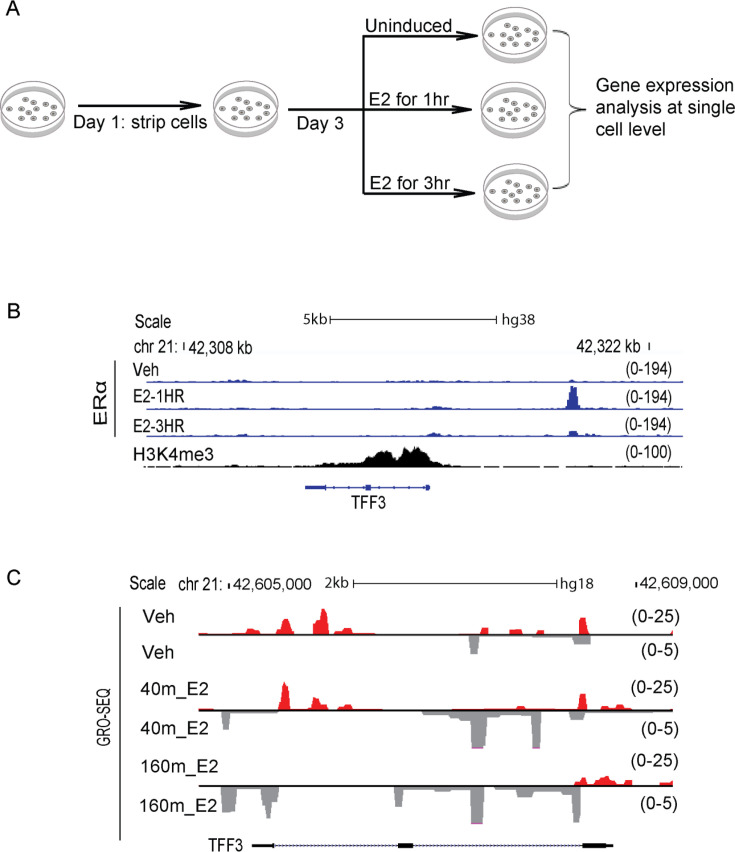
Experimental design. (**A**) Schematic depicting the experimental design. Cells were cultured in complete media for 24 hr followed by stripping for 3 days. Finally, cells were induced for different durations with estradiol (E2)/Vehicle followed by different assays like single molecule RNA FISH (smFISH), 4C-seq, smFISH-IF, etc. (**B**) Zoomed in region around TFF3 gene, UCSC genome browser snapshots showing the binding of ERα and H3k4me3 signal. First, second, and third ERα ChIP-seq tracks are in vehicle-treated, E2-treated for 1 hr and E2-treated for 3 hr in WT cells, respectively. (**C**) UCSC genome browser snapshots showing GRO-seq signal for TFF3 gene. First, second, and third GRO-seq tracks are from vehicle-treated, E2-40m, and E2-160m in WT cells, respectively.

We chose to answer these questions using an integrated approach consisting of single molecule RNA FISH (smFISH), genome-wide conformation capture and sequencing (4C-seq), and perturbation of cis-acting regulatory elements by CRISPR. We identified that TFF1 and TFF3 genes located within the same TAD show distinct and opposing expression profiles over the course of E2-mediated signaling. In agreement with this, the enhancer also showed increased looping interaction with the cognate gene promoter during peak expression at 1 hr of signaling. We identified a potential role of TF-mediated condensates on the up-regulation of TFF1 and down-regulation of TFF3 at the peak of signaling. Hence, we propose that ligand-dependent ERα-protein assemblies can support the expression of the cognate gene in concert with the enhancer that indirectly represses expression of a neighboring gene in the same TAD.

## Results

### ERα binding and ligand-induced gene expression of TFF1 and TFF3 change over the course of estrogen signaling

In order to understand how acute activation of one gene in a TAD affects the transcription of a proximate gene, we chose to study TFF1 and its neighboring gene TFF3 at a 43 kb distance within the same TAD. TFF1 expression is linked with an enhancer located 10 kb downstream (Li et al., 2013; Saravanan et al., 2020; Oh et al., 2021) and the distance between TFF1 enhancer and TFF3 gene is 53 kb (Figure 1A).

17-β-oestradiol exposure leads to ERα binding on regulatory regions, leading to gene activation. Gene transcription of E2-regulated genes was shown to peak at 1 hr and significantly reduced at 3 hr due to the rapid degradation of ERα (Hah et al., 2011). The binding of ERα in the genome also follows this temporal kinetics,where it peaks at 1 hr and reduces at 3 hr post ligand stimulation (Hah et al., 2011; Li et al., 2013; Liu et al., 2014). At the TFF1 locus, the TFF1 enhancer and to some extent, its promoter, were bound by ERα even in the absence of E2 induction, while it increased in strength at these regions and also at various other regions in the locus at 1 hr of ligand stimulation (Saravanan et al., 2020). Notably, the binding at these regions substantially decreased at 3 hr. On the other hand, ERα did not bind on the TFF3 promoter throughout the course of signaling (Figure 1—figure supplement 1B). A single peak of ERα was observed ∼3 kb upstream to TFF3 gene. We have previously shown that estrogen-induced clustered binding of ERα on TFF1 region is associated with its acute activation post-estrogen stimulation (Saravanan et al., 2020). We then tested their expression level by nascent RNA-seq (GRO-seq). We observed a dramatic transcriptional activation of TFF1 at 1 hr, which reduced substantially at 3 hr (Figure 1A). The expression level of TFF3 was comparatively lower and exhibited mild induction at 1 hr which increased further at 3 hr upon signaling (Figure 1—figure supplement 1B). However, qRT-PCR on unspliced TFF3 exhibited upregulation at 1 hr and down-regulation at 3 hr (Figure 1B). We reasoned that these differences in TFF3 could be because of its low baseline expression (Clark et al., 2015; Conesa et al., 2016; Sha et al., 2015; Svensson et al., 2017; Tarazona et al., 2011), and lower yet expression of unspliced transcripts. Therefore, in order to test the relative induction of these genes and their co-regulation, we decided to perform single-cell measurements of RNA (Figure 1—figure supplement 1A) that do not rely on PCR amplification and can provide absolute numbers of TFF1 and TFF3 transcripts in a given cell. Towards this, we chose to employ smFISH (Femino et al., 1998; Haimovich and Gerst, 2018; Kwon, 2013; Raj et al., 2008).

### TFF1 and TFF3 exhibit opposite trends during the E2 signaling time-course

MCF7 cells are hypertriploid to hypotetraploid, and each of the three alleles within a nucleus can behave differently, and smFISH is pre-eminently suited to capturing cell- and allele-specific heterogeneity of expression even for low expressing genes, compared to bulk studies that average over cell populations. Briefly, smFISH allows to visualize single RNA molecules in fixed cells using multiple fluorescently labelled oligonucleotide probes targeted to the RNA of interest and, therefore, it can be used to image the transcription and the localization of multiple gene transcripts at same and different time points after signaling (Femino et al., 1998; Raj et al., 2008).

We designed the smFISH probes targeting the intronic region of TFF1 and TFF3 to measure their nascent transcripts (referred to as InTFF1 and InTFF3 hereon), whereas the exonic probes were used to primarily measure mature mRNA transcripts (referred to as ExTFF1 and ExTFF3 hereon). Intronic probes are particularly suited for investigating nascent transcriptional status at the time of fixation, while probes against mature mRNA reflect on more steady-state levels of functional mRNA due to finite mRNA lifetimes (Skinner et al., 2016). Additionally, the intronic probes can help determine the localization and number of alleles that are transcribing at a given time thus allowing for additional interpretations regarding the expression of multiple genes (Skinner et al., 2016).

**Figure 2.**
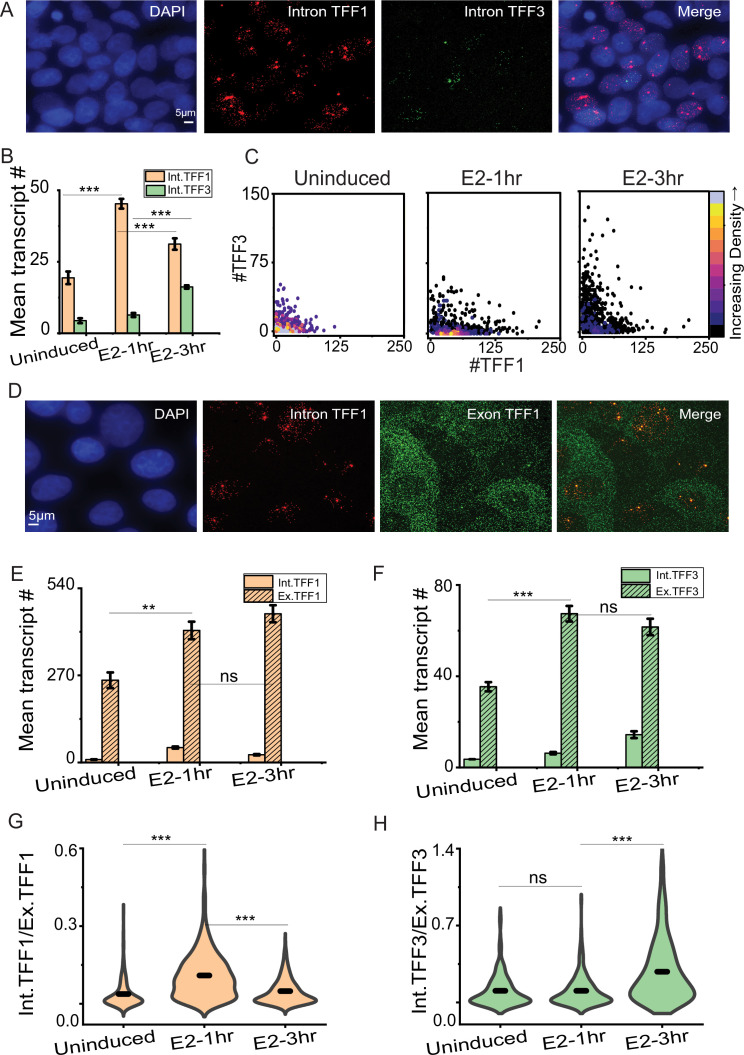
TFF1 and TFF3 expressions show opposite trends during the estradiol (E2) signaling time-course. (**A**) 60 X Representative images from single molecule RNA FISH experiment showing transcripts for TFF1 and TFF3. The probe was designed against the unspliced RNA containing the intronic region. The scale bar is 5 microns. (**B**) The mean RNA numbers are depicted. These are counted using an in-house MATLAB code which uses the DAPI-stained nuclei as the mask to count the RNA present in the nucleus. The graph shows the mean of means from three different repeats of the experiment, and error bars denote SEM (n=665, N=3). *P*-values were calculated by Student’s two-tailed unpaired t-test, and the significance is represented as: *** denotes *p*<0.001, ** denotes *p*<0.01, * denotes *p*<0.05, ns denotes *p*>0.05. (**C**) Scatter plots showing the distribution of InTFF1 and InTFF3 on a cell-by-cell basis (n=665, N=3). The absolute RNA numbers are combined from three different repeats. Density plots have been used to clearly visualize overlapping data points. (**D**) 60 X Representative images from single molecule RNA FISH experiment showing transcripts for InTFF1 and ExTFF1. Scale bar is 5 micrometers. (**E**) The mean RNA numbers for InTFF1 and ExTFF1 are depicted. Separate probes were used to target unspliced (InTFF1) and mature (ExTFF1) RNA. These are counted using an in-house MATLAB code which uses the DAPI-stained nuclei as the mask to count the intronic RNA present in the nucleus and a free-drawn region to designate the cell to count the exonic RNA present in the nucleus as well as the cytoplasm. The graph shows the mean of means from three different repeats of the experiment, and error bars denote SEM (n>360, N=3). *P*-values were calculated by Student’s two-tailed unpaired t-test, and the significance is represented as: *** denotes *p*<0.001, ** denotes *p*<0.01, * denotes *p*<0.05, ns denotes *p*>0.05. (**F**) The mean RNA numbers for InTFF3 and ExTFF3 are depicted. Separate probes were used to target unspliced (InTFF3) and mature (ExTFF3) RNA. The graph shows the mean of means from three different repeats of the experiment, and error bars denote SEM (n>210, N=3). *P*-values were calculated by Student’s two-tailed unpaired t-test, and the significance is represented as: *** denotes *p*<0.001, ** denotes *p*<0.01, * denotes *p*<0.05, ns denotes *p*>0.05. (**G**) Violin plots showing the ratio of intronic to exonic TFF1 counts are depicted. The graph shows the distribution of ratios combined from three different repeats (n>360, N=3). *P*-values were calculated by the Mann-Whitney test, and the significance is represented as: *** denotes *p*<0.001, ** denotes *p*<0.01, * denotes *p*<0.05, ns denotes *p*>0.05. (**H**) Violin plots showing the ratio of intronic to exonic TFF3 counts are depicted. The graph shows the distribution of ratios combined from three different repeats (n>210, N=3). *P*-values were calculated by the Mann-Whitney test, and the significance is represented as: *** denotes *p*<0.001, ** denotes *p*<0.01, * denotes *p*<0.05, ns denotes *p*>0.05. Figure 2—source data 1.Transcript counts from smFISH.

Representative images from the smFISH experiment probing InTFF1 and InTFF3 are depicted (Figure 2A). The smaller foci represent individual intronic/nascent transcripts while the larger foci represent the site of transcription (Raj et al., 2006; Zenklusen et al., 2008). Since MCF7 cells are hypertriploid in nature, we expected to see 1–3 large foci per cell representing sites of transcription. Additionally, we observed many individual transcripts labelled by intronic probes (Figure 2A and D) within the same nuclei (probes against mature RNA are usually more cytoplasmic). This is suggestive of the fact that the transcripts undergo non-co-transcriptional splicing, as the transcripts labeled by intronic probes are localized away from the site of transcription. This is not surprising as several reports have shown that nascent transcripts can undergo splicing well after transcription (Coulon et al., 2014; Drexler et al., 2020; Khodor et al., 2012).

**Figure 2—figure supplement 1.**
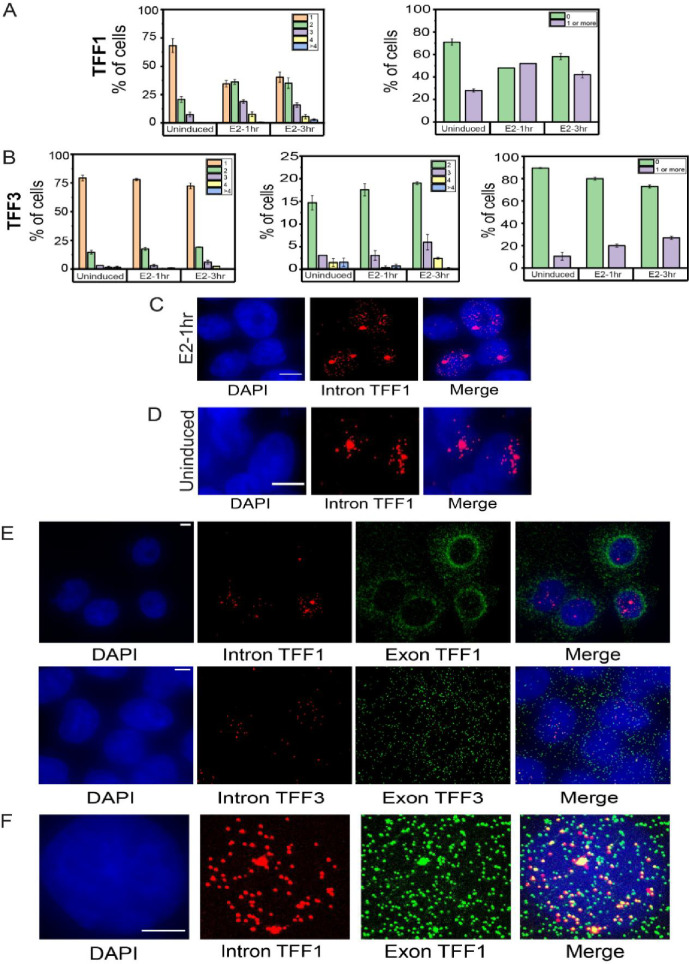
Dynamic induction and RNA localization of TFF1 and TFF3 transcription across cell populations using single molecule RNA (smRNA) FISH. (**A**) Bar graph depicting the percentage of cells with 1, 2, 3, 4, or greater than four sites of transcription for TFF1 (left) is shown. The graph shows the mean of means from different repeats of the experiment, and error bars denote SEM (n>200, N=3). Only the cells with at least one allele firing were counted and cells with no alleles were not included in this. The graph on right shows the number of cells with zero or non-zero number of alleles firing. (**B**) Bar graph depicting the percentage of cells with 1, 2, 3, 4 or greater than four sites of transcription for TFF3 (left) is shown. The graph shows the mean of means from different repeats of the experiment, and error bars denote SEM (n>200, N=3). Only the cells with at least one allele firing were counted and cells with no alleles were not included in this. The graph in the middle shows the number of cells with 2, 3, 4, or greater than four sites of transcription for TFF3. The graph on the right shows the number of cells with zero or non-zero number of alleles firing. (**C**) Images from single molecule RNA FISH experiment showing transcripts for InTFF1 in cells induced for 1 hr with E2. The image shows that when a single allele of TFF1 is firing, the transcripts show a more spatially restricted localization. The scale bar is 5 microns. (**D**) Images from single molecule RNA FISH experiment showing transcripts for InTFF1 in uninduced cells. The image shows that when a single allele of TFF1 is firing and transcription is low, the transcripts show a more spatially restricted localization. The scale bar is 5 microns. (**E**) 60 X Representative images from a single molecule RNA FISH experiment showing transcripts for InTFF1 and ExTFF1 (top) and InTFF3 and ExTFF3 (bottom). The image shows that there is no intronic signal in the cytoplasm, while exonic signals can be found both in the nucleus and the cytoplasm. The scale bar is 5 microns. (**F**) 60 X Representative images from single molecule RNA FISH experiment showing transcripts for InTFF1 and ExTFF1. The image shows that all intronic signals are colocalized with exonic signals, but all exonic signals are expectedly not colocalized with intronic signals, representing more mature mRNA. The scale bar is 5 microns. Figure 2—figure supplement 1—source data 1.Percentage of cell from smFISH data.

**Figure 2—figure supplement 2.**
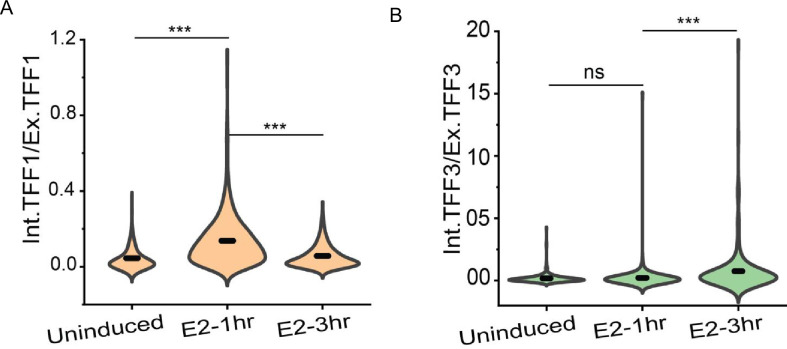
TFF1 and TFF3 expressions show opposite trends during the E2-signaling time-course. (**A**) Violin plot showing the ratio of total intronic to absolute exonic TFF1 counts are depicted. Absolute exonic counts are calculated by subtracting total intronic transcripts from total exonic transcripts. The graph shows the distribution of ratios combined from three different repeats. Error bars denote SEM. *P*-values were calculated by the Mann-Whitney test, and the significance is represented as: *** denotes *p*<0.001, ** denotes *p*<0.01, * denotes *p*<0.05, and ns denotes *p*>0.05. (**B**) Violin plot showing the ratio of total intronic to absolute exonic TFF3 counts are depicted. Absolute exonic counts are calculated by subtracting total intronic transcripts from total exonic transcripts. The graph shows the distribution of ratios combined from three different repeats. Error bars denote SEM. *P*-values were calculated by the Mann-Whitney test, and the significance is represented as: *** denotes *p*<0.001, ** denotes *p*<0.01, * denotes *p*<0.05, and ns denotes *p*>0.05. Figure 2—figure supplement 2—source data 1.Ratio of intronic to exonic counts from smFISH data.

We quantified the number of such transcripts per nuclei as a proxy for ongoing transcription. At 1 hr post E2 induction, TFF1 mean transcript counts increased significantly compared to uninduced, whereas the increment was less pronounced for TFF3 transcripts. In contrast, the mean transcript counts for TFF3 increased significantly at 3 hr post induction while TFF1 transcription showed a decrease at 3 hr compared to 1 hr (Figure 2B). We observed an increase in the number of transcribing sites per cell for both TFF1 and TFF3, as well as in the number of cells showing transcripts compared to the untreated condition (Figure 2—figure supplement 1A, B). Furthermore, TFF1 transcript spots were more spread out in the nucleus that harbored multiple transcribing sites (Figure 2—figure supplement 1C, D). To check the transcriptional status of TFF1 and TFF3 in the same cell, we plotted the transcript counts from individual cells (Figure 2C). The data suggested that the transcript counts for TFF1 increased at 1 hr and cells that showed high counts for TFF1 were likely to have low counts for TFF3 and vice-versa. But the RNA counts for TFF1 decreased at 3 hr and increased for TFF3. Overall, it was evident that the transcriptional profile for these two genes located in the same TAD were negatively correlated as they peaked at different time points. To further confirm this, smFISH using probes targeting both the intronic and exonic transcripts in the same experiment was conducted. Intronic probes represent active transcription, while exonic probes show accumulated mature RNA from past transcriptional events even in the absence of active transcription (Skinner et al., 2016). Representative images from the smFISH experiment probing InTFF1/ExTFF1 and InTFF3/ExTFF3 are depicted (Figure 2D, Figure 2—figure supplement 1E). InTFF1 spots overlapped with ExTFF1 in the nucleus (Figure 2D, Figure 2—figure supplement 1F) and InTFF1 spots were restricted to the nucleus, whereas ExTFF1 were both in the nucleus and the cytoplasm as expected, indicating the specificity of smFISH probes (Figure 2D, Figure 2—figure supplement 1E). We observed that the exonic transcript counts for both TFF1 and TFF3 increased at 1 hr compared to uninduced (Figure 2E and F). Strikingly, at 3 hr, the exonic transcripts for TFF1 continued to increase even while the intronic transcript counts reduced, though the fold increase (1.12±0.02) was less compared to that between uninduced and 1 hr (1.63±0.28), indicating a plateauing of steady-state levels. Exonic transcripts for TFF3 at 3 hr remained comparable to 1 hr while the intronic counts increased suggesting TFF1 transcription reduces at 3 hr, whereas TFF3 expression increases. We reasoned that the ratio of intronic transcript number (InTFF) to exonic transcript number (ExTFF) should represent the status of transcription as an increase in the number of intronic transcripts due to expression would result in a higher ratio while also taking into consideration the number of mature transcripts. As expected, the ratio of intronic transcripts to exonic transcripts also showed that transcription is active at 1 hr for TFF1 as the ratio is higher compared to uninduced and 3 hr (Figure 2G, Figure 2—figure supplement 2A). Contrastingly, the ratio was highest at 3 hr for TFF3 suggesting that active transcription takes place much after the peak of E2-mediated signaling and maximal TFF1 expression (Figure 2H, Figure 2—figure supplement 2B). Indeed, a very recent study has shown that such post-transcriptional splicing can occur for genes and intron dispersal can be expected more commonly for some highly expressed genes (Coté et al., 2023). Therefore, the observation of intronic signal away from the site of transcription as we see for TFF1 and TFF3 is not unexpected (Figure 2A,D, Figure 2—figure supplement 1).

### TFF1 enhancer does not change target promoters during signaling time-course

**Figure 3.**
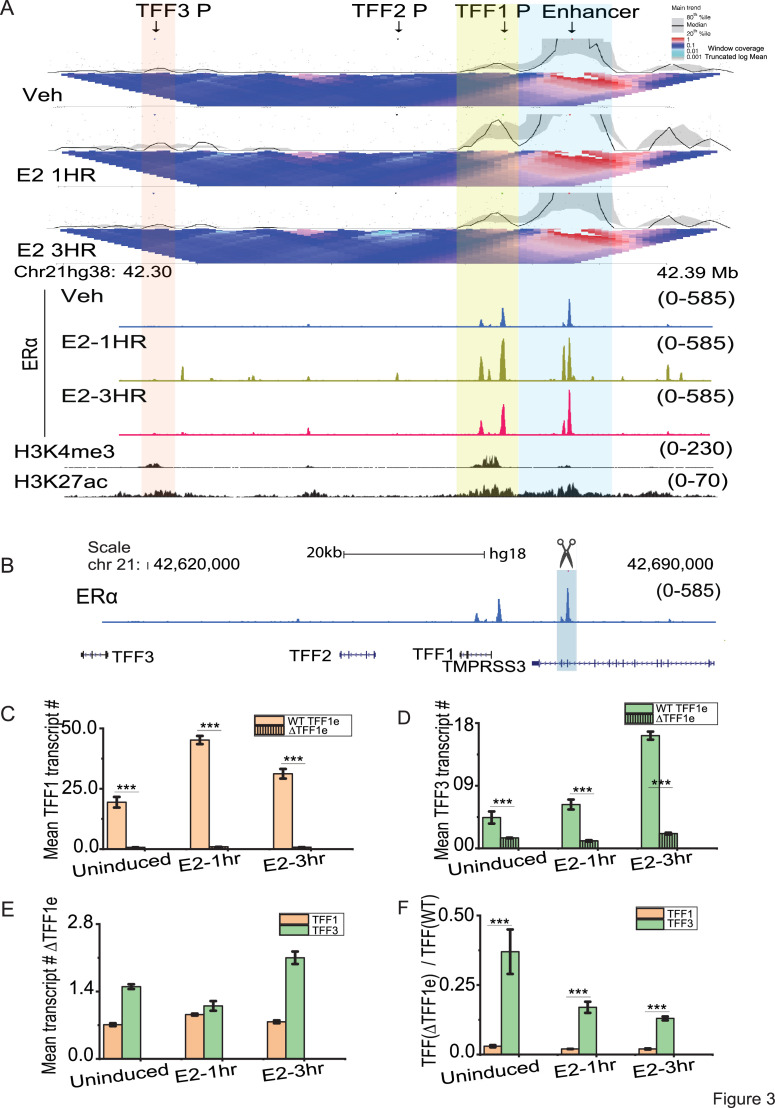
Enhancer looping does not account for the differential expression of TFF1 and TFF3 genes. (**A**) 4C-seq plot at TFF1 enhancer viewpoint, the interaction with the promoter is highlighted in yellow. The plot is overlaid with H3K27ac, ERα ChIP signal, and gene annotations. (**B**) Genome browser snapshot of TFF1 region depicting ERα binding in WT lines. The first, second, and third ERα ChIP-seq tracks are from WT cells that are vehicle-treated, E2-1 hr, and E2-3 hr, respectively. Blue highlighted regions represent the ΔTFF1e region. (**C**) The mean RNA numbers for InTFF1 in WT (unshaded) and ΔTFF1e (shaded) MCF7 cells are depicted. The mean of means are shown, and error bars denote SEM from three repeats (n>650, N=3 for each WT and delete line). *P*-values were calculated by Student’s two-tailed unpaired t-test, and the significance is represented as: *** denotes *p*<0.001, ** denotes *p*<0.01, * denotes *p*<0.05, ns denotes *p*>0.05. (**D**) The mean RNA numbers for InTFF3 in WT (unshaded) and ΔTFF1e (shaded) MCF7 cells are depicted. The mean of means are shown, and error bars denote SEM from three repeats (n>650, N=3 for each WT and delete line). *P*-values were calculated by Student’s two-tailed unpaired t-test, and the significance is represented as: *** denotes *p*<0.001, ** denotes *p*<0.01, * denotes *p*<0.05, ns denotes *p*>0.05. (**E**) The mean RNA numbers for InTFF1 and InTFF3 in ΔTFF1e MCF7 cells are depicted. The mean of means are shown, and error bars denote SEM from three repeats (n>880, N=3). *P*-values were calculated by Student’s two-tailed unpaired t-test, and the significance is represented as: *** denotes *p*<0.001, ** denotes *p*<0.01, * denotes *p*<0.05, ns denotes *p*>0.05. (**F**) Ratio of InTFF in WT MCF7 to InTFF in ΔTFF1e MCF7 are depicted. The ratio was obtained by dividing the absolute RNA counts of the WT line by delete lines performed on different days but in the same order (replicate one of WT divided by replicate one of ΔTFF1e). The mean of means are shown, and error bars denote SEM from three repeats (n>650, N=3 for each WT and delete line). *P*-values were calculated by Student’s two-tailed unpaired t-test, and the significance is represented as: *** denotes *p*<0.001, ** denotes *p*<0.01, * denotes *p*<0.05, ns denotes *p*>0.05. Figure 3—source data 1.Data for 4C (Figure 3A) and smFISH (Figure 3C–F).

In order to identify the molecular players behind the differential expression of these two genes that are in the same TAD, we asked if the ERα-bound enhancer downstream to the TFF1 gene loops with TFF1 at 1 h, and with TFF3 at 3 hr. This enhancer acutely activates TFF1 at 1 hr post-estrogen stimulation (Saravanan et al., 2020; Oh et al., 2021). We interrogated the looping using enhancer as a viewpoint by 4C-seq in uninduced, 1 hr and 3 hr post E2 stimulation. We observed robust interactions between enhancer and TFF1 promoter at 1 hr post-induction, which reduced at 3 hr. On the other hand, its interaction with the TFF3 promoter exhibited very low counts in uninduced as well as E2-induced conditions at both time points and in both replicates (Figure 3A, Figure 3—figure supplement 1). This suggests that the looping of enhancer potentially induces the expression of TFF1 gene at 1 hr, and loss of interactions at 3 hr results in weak TFF1 transcription. However, the lack of interactions between enhancer and TFF3 did not explain the gain of TFF3 expression at 3 hr post-ligand stimulation.

**Figure 3—figure supplement 1.**
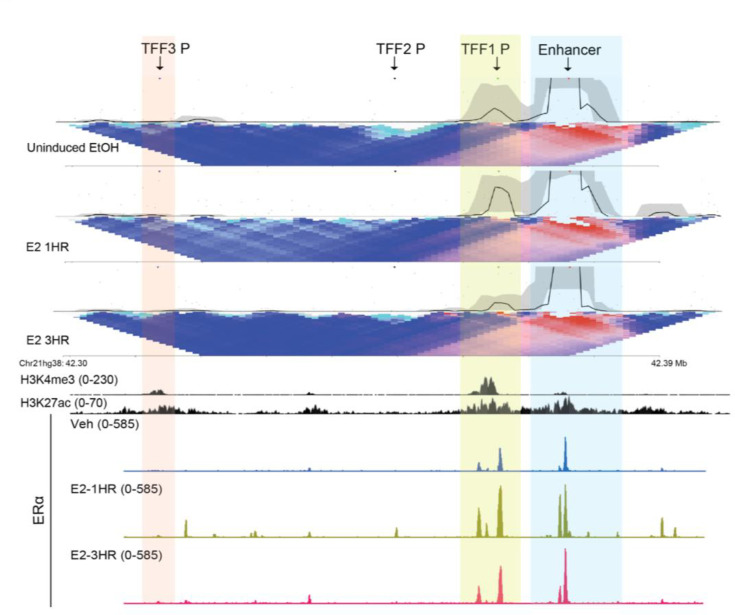
TFF1 enhancer does not change target promoters during signaling time-course. 4C-seq plot at TFF1 enhancer viewpoint, the interaction with the promoter is highlighted in yellow. The plot is overlaid with H3K27ac, ERα ChIP signal, and gene annotations. There is no substantial interaction between the enhancer and TFF3 locus at any time point, while the interaction between the enhancer and TFF1 locus increases at 1hr and decreases at 3hr. Figure 3—figure supplement 1—source data 1.Data from 4C, replicate 2.

This could mean that enhancer interaction is critical for TFF1 expression, but is less important for TFF3 expression. Thus, TFF1 expression should be affected more severely upon deletion of the enhancer than TFF3. To test this, we investigated the expression of TFF1 and TFF3 in MCF-7 cells where the enhancer downstream to TFF1 was homozygously deleted using CRISPR-Cas9 (referred to as the ΔTFF1e from hereon) (Figure 3B; Saravanan et al., 2020). Using smFISH, we quantified the intronic transcripts for TFF1 and TFF3 in the ΔTFF1e cells, compared to WT cells. We observed that the mean number of TFF1 transcripts was reduced drastically in the ΔTFF1e compared to the WT (Figure 3C). The reduction was far more substantial (49.23±2.6 at 1 hr and 40.75±7.8 at 3 hr) for TFF1 than TFF3 (6.03±1.8 at 1 hr and 7.6±0.7 at 3 hr) (Figure 3D). The absolute transcript counts for TFF1 and TFF3 in the ΔTFF1e cells have also been shown for clearer visualization (Figure 3E), as these are obscured when compared to WT. To get a sense of fold changes, we took a ratio of mean transcript counts in WT cells to ΔTFF1e cells at each time point. We observed that the drop in gene transcripts between WT and ΔTFF1e were several folds higher for TFF1 compared to TFF3 (Figure 3F). This suggests that enhancer deletion has a more robust impact on the transcription of TFF1 compared to TFF3. Nonetheless, TFF3 was also affected even though it does not loop with the enhancer. This is in accordance with the 4C-seq data where we observed prominent looping between the enhancer and TFF1 but less so with TFF3. These results suggest that the enhancer plays a more important role in the expression of the primary gene while it has less impact on a gene located more distally but within the same TAD during the course of signaling.

### Levels of ERα in the nucleus dictate the extent of TFF1 and TFF3 inductions

**Figure 4.**
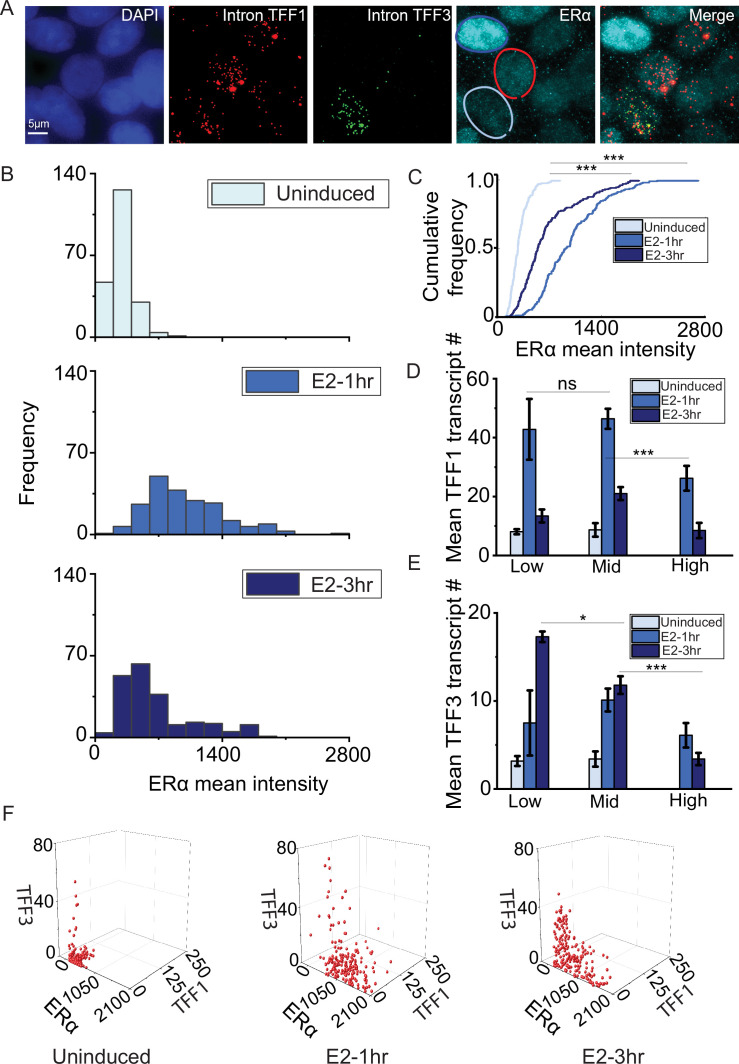
Nuclear levels of ERα dictate the extent of TFF1 and TFF3 expression. (**A**) Representative images showing smFISH for InTFF1 and InTFF3 in combination with immunofluorescence for ERα (along with chromatin retention assay). The red circle denotes a cell with high ERα and low TFF1 and TFF3, the blue circle denotes a cell with medium ERα, high TFF1, and low TFF3 while the green circle denotes a cell with low ERα and high TFF3. The scale bar is 5 micrometres. (**B**) Histogram representing the distribution of ERα mean intensities in cells under uninduced, E2-1 hr, and E2-3 hr conditions (n=210). Intensities at 1 hr are the highest while they shift to the left at 3 hr and are lowest in uninduced cells. This is plotted from one experimental repeat out of three repeats as ERα intensities will vary from one immunofluorescence experiment to another. (**C**) Cumulative histogram representing the distribution of ERα mean intensities in cells under uninduced, E2-1 hr, and E2-3 hr conditions. *P*-values were calculated by the Mann-Whitney test and the significance is represented as: *** denotes *p*<0.001, ** denotes *p*<0.01, * denotes *p*<0.05, ns denotes *p*>0.05. (**D**) ERα intensities were sorted into three categories namely low (intensities between 0–450 A.U.), mid (intensities between 450–1200 A.U.), and high (intensities between 1200–2100 A.U.). The mean and SEM of transcript count for InTFF1 in the three categories under uninduced, E2-1 hr, and E2-3 hr was plotted. Low and mid categories show the highest TFF1 mean. *P*-values were calculated by the Mann-Whitney test and the significance is represented as: *** denotes *p*<0.001, ** denotes *p*<0.01, * denotes *p*<0.05, ns denotes *p*>0.05. This is plotted from one experimental repeat out of three repeats as ERα intensities will vary from one immunofluorescence experiment to another. (**E**) ERα intensities were sorted into three categories namely low (intensities between 0–450 A.U.), mid (intensities between 450–1200 A.U.), and high (intensities between 1200–2100 A.U.). The mean and SEM of transcript count for InTFF3 in the three categories under uninduced, E2-1 hr, and E2-3 hr was plotted. Low category shows the highest TFF3 mean. *P*-values were calculated by the Mann-Whitney test and the significance is represented as: *** denotes *p*<0.001, ** denotes *p*<0.01, * denotes *p*<0.05, ns denotes *p*>0.05. This is plotted from one experimental repeat out of three repeats as ERα intensities will vary from one immunofluorescence experiment to another. (**F**) 3D plot representing the distribution of ERα, InTFF1, and InTFF3 on a cell-by-cell basis shows that cells with lower levels of ERα show higher counts for InTFF3. This is plotted from one experimental repeat out of three repeats as ERα intensities will vary from one immunofluorescence experiment to another. Figure 4—source data 1.Data is from smFISH (TFF1 and TFF3) and Immunofluorscence (Estrogen receptor alpha).

After ruling out enhancer looping as the determinant of differential expression, we looked for other candidates that could regulate the differential gene expression. As discussed above, globally, E2-mediated gene expression is known to peak at 1 hr after stimulation and drop significantly by 3 hr (Hah et al., 2011). Upon ligand stimulation, ERα translocates into the nucleus, increasing mean ERα intensity in the nucleus which is high at 1 hr and then decreases significantly by 3 hr due to degradation. These changes in intensities have been captured using immunofluorescence for ERα (Saravanan et al., 2020). We tested if the intensities of ERα in individual cells correlate with the expression of TFF1 and TFF3 at 1 hr and 3 hr of E2 signaling. Towards this, we combined smFISH with immunofluorescence for ERα. To improve contrast for the ERα signal, we additionally performed a chromatin retention assay to get rid of any chromatin unbound ERα (Figure 4A). The representative images show that the cells with very high levels of nuclear ERα (blue circle) exhibited low counts of both TFF1 and TFF3. In contrast, the cells with medium levels of ERα (red circle) possessed higher TFF1 than TFF3. Similarly, the cells with the lower levels of ERα (gray circle) showed higher TFF3 expression as compared to TFF1 (Figure 4A). Histograms depicting the ERα mean intensities across individual cells, showed that the nuclear level of ERα increases post 1 hr of induction and then goes down at 3 hr (Figure 4B and C), similar to TFF1 expression (Figure 4D).

**Figure 4—figure supplement 1.**
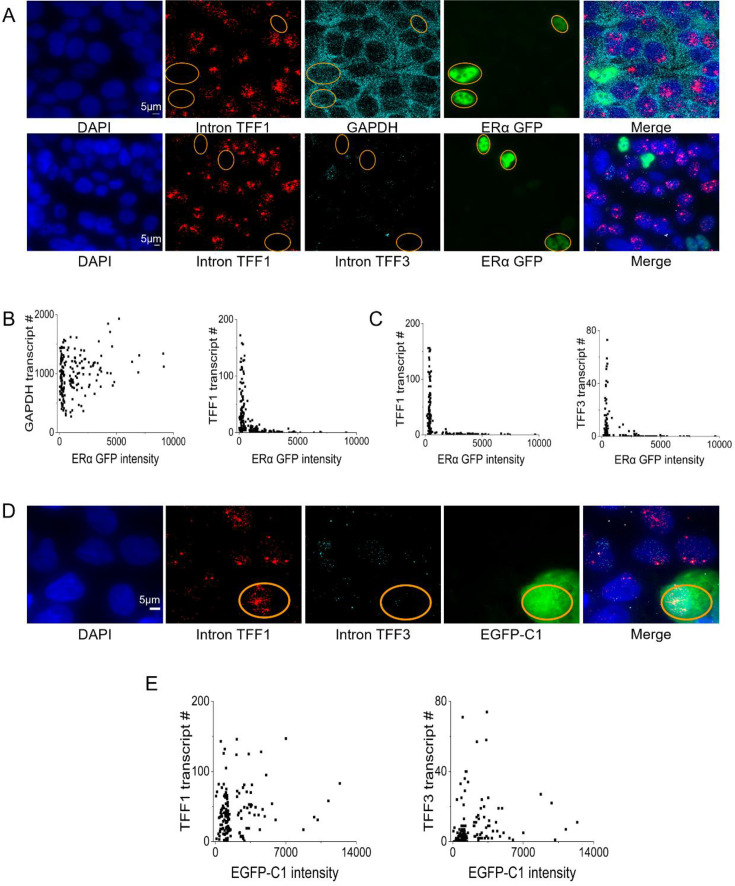
Levels of ERα in the nucleus dictate the extent of TFF1 and TFF3 inductions. (**A**) Representative images from smFISH experiments showing InTFF1 and GAPDH (top panel) and InTFF1 and InTFF3 (bottom panel) in cells overexpressing ERα-GFP. Yellow circles show cells that are GFP positive and the transcripts associated with them. Note the visibly fewer TFF1, and TFF3 transcripts in the ERα-GFP-positive cells while GAPDH transcripts remain indistinguishable. This shows that ERα-GFP overexpression specifically affects the transcription of E2-regulated genes like TFF1 and TFF3 and not a housekeeping gene like GAPDH. This is further quantified in B and C. (**B**) Scatter plots showing the distribution of ERα-GFP intensities with GAPDH or InTFF1 transcript counts on a cell-by-cell basis from the experiment in the first row of A. Cells with high ERα-GFP expression can have high GAPDH expression but such cells necessarily have low TFF1 counts. (**C**) Scatter plots showing the distribution of ERα GFP intensities with InTFF1 or InTFF3 counts on a cell-by-cell basis from the experiment in the second row of A. As in B, high ERα-GFP expression leads to low expression of E2-regulated genes like TFF1 or TFF3. (**D**) Representative images showing smFISH for InTFF1 and InTFF3 in cells overexpressing EGFP-C1. Yellow circles show cells that are GFP positive and the transcripts associated with them. Just expressing EGFP in the same backbone as the ERα-GFP plasmid does not downregulate TFF1, and TFF3 expression; indicating that the downregulation effect is specific to ERα overexpression and not a generic effect of cell transfection. This is quantified in E. (**E**) Scatter plots showing the distribution of EGFP-C1 intensities with InTFF1 or InTFF3 transcript counts on a cell-by-cell basis. There is no obvious correlation between the levels of EGFP-C1 and TFF1 or TFF3, and a cell with high EGFP levels may well have large TFF1 or TFF3 transcript counts. Figure 4—figure supplement 1—source data 1.Data is from smFISH (TFF1, TFF3 and GAPDH) and ERa-GFP and GFP alone.

To further corroborate this, we parsed the ERα population cells into three categories namely low, medium, and high within the cells imaged at 1 and 3 hr. We plotted the mean counts of TFF1 in each of these bins at different time points and observed that the mean count was higher in the mid-category (Figure 4D). While for TFF3, the mean count was significantly higher in the low bin (Figure 4E). The transcript counts for TFF1 and TFF3 against ERα intensities on a cell-by-cell basis (Figure 4F) also showed this feature where very high levels of ERα in the nucleus were not conducive to the expression of either gene (Figure 4F). To test if ERα had a causal role in intensity based expression of TFF1 and TFF3, we increased the levels of ERα by overexpression of ERα-GFP (Figure 4—figure supplement 1A). The representative images from the smFISH experiment in cells overexpressing ERα-GFP confirmed that TFF1 and TFF3 were downregulated in transfected cells while these genes were not perturbed in non-transfected cells in the neighborhood (Figure 4—figure supplement 1A, C). Meanwhile, the cells overexpressing ERα-GFP do not show any impairment in the expression of GAPDH (housekeeping gene) (Figure 4—figure supplement 1A, B). As another control, we transfected the cells with EGFP-C1 (same backbone as ERα-GFP construct) and observed no effect on TFF1 or TFF3 (Figure 4—figure supplement 1D, E). The data suggest that loss of TFF1 and TFF3 expression upon ERα overexpression was not a general effect of transfection stress but rather specific to ERα overexpression. These results indicate that high nuclear levels of ERα can be detrimental to the expression of genes it regulates. This could be due to the widespread condensate formation, which in turn could sequester the transcriptional protein complexes and competitively abrogate transcription across multiple loci. Thus, the global level of ERα in the nucleus can predict the transcriptional status of specific genes. The binding of ERα at 1 and 3 hr is proportional to its nuclear levels (Figure 3A), suggesting its overexpression would lead to more binding in the genome, which is detrimental to gene expression.

### 1,6 HD exposure down-regulates TFF1 but supports TFF3 expression

**Figure 5.**
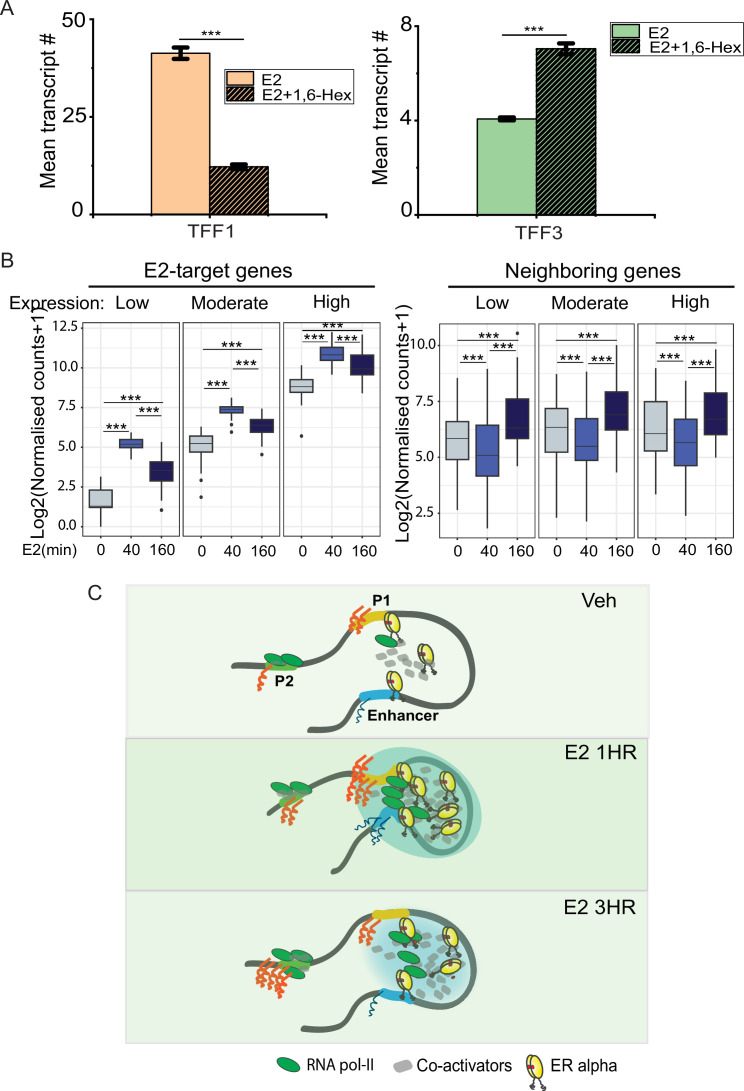
1,6 HD exposure down-regulates TFF1 but supports TFF3 expression. (**A**) Mean transcript counts for InTFF1 and InTFF3 in control, and 3% 1,6-Hexanediol treated cells post 30 min of E2- induction. The mean of means are shown, and the error bars denote SEM from three repeats (n>880, N=3). *P*-values were calculated by Student’s two-tailed unpaired t-test, and the significance is represented as: *** denotes *p*<0.001, ** denotes *p*<0.01, * denotes *p*<0.05, ns denotes *P*>0.05. (**B**) Boxplots showing DESeq2 normalized counts for low expressing, moderately expressing, and highly-expressing genes in the vehicle, E2-40m, and E2-160m, respectively (left). Boxplots showing DESeq2 normalized counts for genes near low expressing, moderately expressing, and highly expressing genes in the vehicle, E2-40m, and E2-160m, respectively (right). The *p*-values in the boxplots were calculated using the Wilcoxon rank-sum test. The boxplots depict the minimum, first quartile, median, third quartile, and maximum values, along with outliers. (**C**) Model depicting the signaling under uninduced, E2-1 hr, and E2-3 hr conditions – First, activation of target gene loci (like TFF1) occurs by ligand-dependent induction. During the active phase (1 hr), liganded ERα binds on enhancer and promoter. Together, these elements interact in 3D, manifesting as ERα punctae, which results in robust expression of target genes, and the sequestration of transcriptional machinery. At 3 hrs, as nuclear ERα decreases, the transcriptional machinery becomes available to other nearby promoters leading to increase in gene transcription at those loci. Figure 5—source data 1.smFISH counts for TFF1 and TFF3 upon E2 induction with and without 1,6HD.

The data obtained from combined smFISH and ERα immunofluorescence indicates that ERα could be a determining factor in controlling the differential gene expression of TFF1 and TFF3. Existing literature indicates that ERα-mediated condensate plays a role in E2-induced gene expression (Boija et al., 2018; Nair et al., 2019; Sabari et al., 2018; Saravanan et al., 2020). It led us to hypothesize that ERα-mediated condensate at the TFF1 locus could be sequestering all the factors required for active transcription, thus preventing activation of the TFF3 locus at the active phase of E2 signaling. To test this, we treated the cells with 3% 1,6-hexanediol for 5 min, which is known to disrupt LLPS (Gamliel et al., 2022). Following the treatment, we performed smFISH to look at the transcription of TFF1 and TFF3 in the same cell and observed a dramatic reduction in TFF1 transcripts, whereas a statistically significant increase in TFF3 was noted (Figure 5A). Since ERα forms condensate only after estrogen stimulation, 1,6-hexanediol had no effect on TFF1 in the absence of E2 signaling (Figure 5—figure supplement 1). Together, these results suggest that the functional loss of TFF1 promoter transcription potentially due to the dissolution of ERα condensate allowed the TFF3 promoter in the neighborhood to gain access to transcriptional machinery, leading to its upregulation. In order to test the generality of this observation, beyond the TFF1 and TFF3 locus, we divided E2-responsive genes into three categories, low, moderate, and high, based on their expression upon E2-40m stimulation. We observed a significant increase at 40 m and down-regulation at 160 m when the binding of ERα is reduced in the genome (Figure 5B). Next, we tested the expression of their nearby upregulated genes at 160 m compared to 40 m post-E2 stimulation. The nearby genes are within a distance of 1 Mb from the E2-responsive genes, which is approximately the average size of a TAD. Indeed, we observed a significant upregulation of nearby genes at 160 m when the expression of highly induced genes dropped. Additionally, the expression of these neighboring genes was reduced at the peak of signaling (Figure 5B), showing that at 40 m of signaling, the acute activation of primary genes and sequestration of transcription machinery by these genes leads to the loss of expression of nearby genes.

**Figure 5—figure supplement 1.**
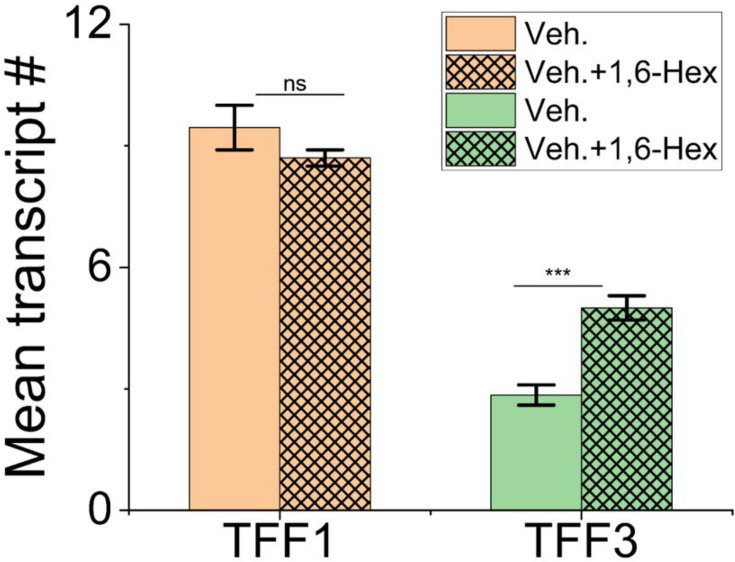
LLPS perturbation down-regulates TFF1 but supports TFF3 expression. Mean transcript counts for InTFF1 and InTFF3 in control, and 3% 1,6-hexanediol-treated cells in the absence of E2-induction show no difference in TFF1 counts on 1,6-hexanediol treatment. The bar denotes the mean of means from two repeats, and the error bars denote SEM. *P*-values were calculated by Student’s two-tailed unpaired t-test, and the significance is represented as: *** denotes *p*<0.001, ** denotes *p*<0.01, * denotes *p*<0.05, ns denotes *p*>0.05. Figure 5—figure supplement 1—source data 1.smFISH counts for TFF1 and TFF3 without E2 induction (with and without 1,6HD).

## Discussion

smFISH allowed us to simultaneously capture allele-level transcription of two genes, TFF1 and TFF3, located in the same TAD, at single-cell level during the peak and fall of signaling. We were able to capture the anti-correlated expression of these two genes, revealing intricate regulatory feedback between acutely activated enhancer-dependent gene, TFF1, which caused the dysregulation of the non-enhancer targeted gene, TFF3, within the same TAD (Figure 5B).

Our data suggests that while potential condensate formation on enhancer allows robust activation of the target gene, however, it negatively impacts the expression of other neighboring genes possibly due to the sequestration of transcriptional machinery from these genes. When the condensates dissolve, the locally enriched transcriptional machinery is available to the other loci, allowing for increased transcription of these genes that are not the direct target of enhancers (Figure 5C). Forced dissolution of condensates by 1,6 HD allows the expression of non-enhancer target genes in the cells, although at low levels. The data also suggests that, globally, the genes next to ligand-induced genes are upregulated only at the fall of signaling as an indirect consequence of excess polymerase availability in the neighborhood.

### Non-enhancer target genes are also regulated indirectly in the same TAD

ERα binding strength increases at the TFF1 enhancer at 1 hr of ligand stimulation (Saravanan et al., 2020). Such estrogen-induced binding of ERα leads to robust ligand-induced activation of TFF1 gene. While TFF3 promoter remains unbound by ERα (Figure 3A) and does not interact with the TFF1 enhancer throughout the course of signaling. These data suggest that TFF3 expression is both ERα and enhancer independent. Despite non-dependence, TFF3 expression was mutually exclusive to TFF1 expression and was negatively impacted in the absence of the enhancer. The latter could be due to the enhancer-mediated repositioning of the entire TAD that benefited the expression of TFF3 and not due to the direct interaction between the enhancer and the TFF3 gene.

The mean expression of TFF1 was many fold higher than TFF3, thus explaining its dependence on an active upstream enhancer. The enhancer of TFF1 interacts with the gene at the peak of signaling and shows some loss of interaction at 3 hr when signaling recedes. As stated above, the enhancer does not interact with TFF3, however, the enhancer forms a loop with TFF3 upon deletion of the TFF1 promoter (Oh et al., 2021). The ERα condensates are formed at the TFF1 locus upon signaling (Saravanan et al., 2020), which is reduced upon enhancer deletion, suggesting its causal role in condensate formation. Corroborating with this, the enhancer of TFF1 engages in the recruitment of megadalton complex (Liu et al., 2014) in MCF-7 cells, which could facilitate phase-separation (Nair et al., 2019; Saravanan et al., 2020). The cells that expressed TFF1 did not express TFF3 as efficiently, suggesting Pol2 was potentially sequestered from the TFF3 promoter. However, at 3 hr when liganded ERα degrades leading to dissolution of potential condensates on enhancer, TFF3 expression increased, suggesting a local redistribution of active transcription machinery to other genes within the same chromatin domain. Even at 1 hr, when condensates were perturbed by 1,6 HD, the expression of TFF3 increased indicating the access to Pol2 pool by TFF3 promoter. The data explains the negative correlation between TFF1 and TFF3 expression at the peak of signaling and robust activation of TFF3 at 3 hr when TFF1 expression decreased at single cell level. The presence of significant numbers of TFF1 nascent RNA in the nucleus in our data could also be due to a time lapse between transcription and splicing, as seen for the genes that are early responders during signaling (Zambrano et al., 2020).

### High and low levels of ERα were detrimental to enhancer-mediated activation of TFF1

We observe that TFF1 expression is the highest in cells with medium levels of ERα, while it shows low expression in cells with low or high levels of ERα (Figure 4D and F). This points to an inverted U-shaped response of TFF1 to ERα, which is a nonmonotonic response to estrogen, as has been shown (Lebedeva et al., 2012; Vandenberg et al., 2012). A nonmonotonic response can be described as an inverted U-shaped curve with maximal response occurring in the middle and reducing substantially at very low or very high doses (Vandenberg et al., 2012). Furthermore, we observe that for TFF3, the maximal expression occurs at low levels of ERα (Figure 4E and F). This could be indicative of a response such that a low concentration is enough to meet the threshold for expression beyond which all other higher doses attenuate the expression (Lebedeva et al., 2012; Vandenberg et al., 2012). This could also suggest that ERα levels have a varied effect on different genes and their expression depending on the extent of reliance of the gene on ERα for its expression. This phenomenon, called the prozone effect, is characterized by the inhibition of multimeric complexes when one of the constituents of the complex are present at very high concentrations as has been shown for TFF1 (Lebedeva et al., 2012). In the context of condensates on TFF1 gene, we speculate that very low levels of TF may not reach or exceed the critical concentration required for LLPS (for TFF1), but this poor transcriptional state of TFF1 could be favourable for TFF3. Alternatively, a very high concentration of the TF could result in the formation of highly dense homogenous condensates, effectively reducing transcription in a situation akin to the prozone effect (Chong et al., 2022; Lebedeva et al., 2012; Ryu et al., 2024).

Interestingly, the levels of ERα do not determine the expression of other E2 target genes like GREB1 and MYC Stossi et al., 2020; however, we observed that overexpression of ERα causes downregulation of TFF1 (Figure 4—figure supplement 1A) and this is in agreement with what has been observed for the expression status of TFF1 in cell line with high ERα compared to cell lines with low ERα (Lebedeva et al., 2012).

ER is known to be expressed in nearly 70% of breast cancers, and many of these are treated using estrogen receptor modulators and degraders (Arnesen et al., 2021). Therefore, understanding the effect of ERα on the expression of estrogen-responsive genes depending on whether they are target genes or non-target genes is of therapeutic importance as several non-target genes have been shown to be differentially regulated in ER-mutant breast cancer cells (Arnesen et al., 2021).

Together, our study underscores the indirect effects of ligand-induced chronic gene transcription that is dependent on enhancer activation and possible TF-condensate within a TAD.

## Materials and methods

### Cell culture

MCF-7 cells (original line procured from ATCC (RRID:CVCL_0031); WT scramble control and ΔTFF1e were generated in Saravanan et al., 2020) were cultured in non-stripping media consisting of DMEM (Gibco, 12100–046) supplemented with 10% FBS (Gibco, 16000–044) and 1% Penicillin-Streptomycin-Glutamine (Gibco, 10378–016) in a 5% CO2 humidified incubator at 37 °C (unstripped MCF-7). MCF-7 cell lines were tested to be estrogen-responsive and negative for mycoplasma contamination. Cells were passaged and seeded into glass bottom dishes in non-stripping media for 24 hr and allowed to reach 60–80% confluency. These cells were then hormone-stripped for three days in stripping media containing phenol red-free DMEM (Gibco, 21063–029) supplemented with 5% charcoal–stripped FBS (Gibco,12676029) and maintained in the humidified incubator at 37 °C (stripped MCF-7).

In order to induce the estrogen transcriptional responses, on the third day cells, were treated with β-estradiol (E2758, Sigma-Aldrich) at 100 nM concentration for various periods as mentioned in the respective Figures. For untreated control, cells were either treated with equal microliters of ethanol on the third day or with ERα inhibitor ICI182780 (1047, Tocris Biosciences) at 100 nM concentration for 24 hr after two days of stripping.

For perturbation of transcriptional condensates in E2-induced condition, cells were incubated with media containing 3% 1,6-hexanediol for 5 min (after 30 min of E2 treatment) followed by removal of 1,6-hexanediol and recovery for 25 min in E2-containing media.

For experiments with overexpression of EGFP- ERα or EGFP, cells were transfected 24 hr before E2 induction using X-tremeGENE HP DNA Transfection Reagent (XTGHP-RO) in stripping media.

### Probe for smFISH

Probes were designed, custom-made, and tagged with indicated fluorophores (Quasar 570 or Cal Fluor 610) by Biosearch Technologies (https://www.biosearchtech.com/products/rna-fish). Probes were made to target the intronic or exonic regions of the TFF1 and TFF3 genes. Probe sequences can be found in Supplementary file 1a-d in supplementary materials.

### Chromatin retention assay and smFISH

For smFISH experiments without chromatin retention assay cells, the cells were treated with estradiol or vehicle control for indicated times. Following this, the cells were washed with nuclease-free 1 X PBS (Ambion, AM9624) twice. This was followed by fixation using 4% paraformaldehyde (PFA, Sigma, P6148) in 1 X PBS for 10 min at room temperature. The fixative was removed and cells were washed twice with 1 X PBS. The cells were permeabilized using 70% ethanol at 4 °C overnight. On the next day, the permeabilizing agent was removed and the cells were washed twice with 1 X PBS. The cells were washed once with a wash buffer (10% Formamide (Ambion, 9342) and 2 X SSC (Ambion, AM9763) in nuclease-free water) for 5 min at room temperature. The wash buffer was aspirated and the cells were incubated with a hybridization mix (100 μl of mix contains 10% formamide, 89 μl hybridization buffer, and 1 μl each of indicated probes) overnight at 37 °C in a humid chamber. The next day, the solution was removed and cells were washed with the wash buffer at 37 °C for 30 min. To stain the nuclei, DAPI (Invitrogen, D1306; 2 μg/ml) in wash buffer was added to the cells and incubated for 10 min at 37 °C. The cells were then washed with 2 X SSC for 5 min at 4 °C. The solution was removed and the cells were covered with a few drops of the mountant Vectashield (Vector Labs). The plates were imaged after at least 1 hr.

For smFISH experiments with chromatin retention assay cells were treated with estradiol or vehicle control for indicated times. Following this, the cells were treated with CSK buffer (consisting of 10 mM PIPES buffer, 100 mM NaCl, 3 mM MgCl2, 300 mM sucrose, and 0.7% Triton-X 100) for 15 min at room temperature. After this, the cells were washed with nuclease-free 1 X PBS twice. This was followed by fixation using 4% paraformaldehyde in 1 X PBS for 10 min at room temperature. The fixative was removed and cells were washed twice with 1 X PBS. The cells were permeabilized using 0.3% Triton-X 100 (Sigma, T8787) in 1 X PBS for 10 min at room temperature. The permeabilizing agent was aspirated and the cells were washed twice with 1 X PBS. This was followed by a washing step using the wash buffer for 5 min at room temperature. Following this, the cells were incubated with the hybridization mix to which the antibody of interest was also added at appropriate dilution. The next day, the hybridization mix containing probes and antibodies was removed. Cells were washed twice with 1 X PBS. Then the cells were incubated with 1 X PBS containing a secondary antibody against the antibody of interest at room temperature for 2 hr. This was followed by incubation with the wash buffer at 37 °C for 30 min. To stain the nuclei, DAPI in wash buffer was added to the cells and incubated for 10 min at 37 °C. The cells were then washed with 2 X SSC for 5 min at 4 °C. The solution was removed and the cells were covered with a few drops of the mountant Vectashield. The plates were imaged after at least 1 hr.

### Antibody staining/immunofluorescence

The primary antibody against ERα (Santa Cruz, sc8002(F10)) was added to the hybridization mix at the dilution of 1:400 and incubated overnight at 37 °C. The next day, an anti-mouse Alexa Fluor 488 secondary antibody (Invitrogen, A11029) was added to 1 X PBS at the dilution of 1:1000. Cells were incubated for 2 hr followed by the continuation of the smFISH protocol as indicated above.

### Image acquisition

The plates were imaged on an Olympus IX83 inverted widefield fluorescence microscope with a Retiga 6000 CCD monochrome camera (QImaging). The images were acquired using a 60 X, 1.42 N.A. oil immersion objective or a 100 X, 1.4 N.A. oil immersion objective. The z-step size was 0.3 μm and 35 slices were acquired. The resolution at which the images were acquired is 2752×2208. Narrow band-pass filters were used to distinguish the signal from Quasar 570 and Cal Fluor 610 labeled probes (ChromaTechnology- 49309 and 49310).

### Image analysis and representation

The images of mRNA channels were subjected to rolling ball background subtraction across the entire Z-stack using Fiji. For representative images, the stacks were Z-projected in Fiji. Transcripts were counted using tools courtesy of Arjun Raj (https://rajlab.seas.upenn.edu/) using a MATLAB (Mathworks) script originally developed in an earlier work (Raj et al., 2008). A nuclear mask was used to count the intron-containing RNA present in the nucleus (InTFF), and another mask for the whole cell to count the mature mRNA, detected by exonic probes (ExTFF), that are present in the nucleus as well as the cytoplasm. The mean intensity for the ERα immunofluorescence channel was also quantified using the MATLAB script on a cell-by-cell basis using the nuclear mask.

### Graphing and statistics

The graphs were plotted using Python 3, MATLAB, and Origin Pro and edited using Adobe Photoshop. To perform a student’s t-test for data combined from three repeats, GraphPad (https://www.graphpad.com/quickcalcs/ttest1/) was used. Non-parametric tests were performed in case of single cell data. For this, the Mann-Whitney test was used. Significance is represented as: *** denotes *p*<0.001, ** denotes *p*<0.01, * denotes *p*<0.05, ns denotes *p*>0.05. The specific test used has been mentioned in the relevant figure legends.

### Circular chromatin conformation capture-seq

4 C was performed as per the protocol described in van de Werken et al., 2012 with minor variations. MCF7 cells were fixed with fresh formaldehyde (1.5%) and quenched with glycine (125 mM) followed by washes with ice-cold 1XPBS (2 X) and scraped, pelleted, and stored at –80 °C. Lysis buffer [Tris-Cl pH 8.0 (10mM), NaCl (10 mM), NP-40 (0.2%), PIC (1 X)] was added to the pellets and homogenized by Dounce homogenizer (20 stroked with pestle A followed by pestle B). The 3 C digestion was performed with DpnII (200 units, NEB) and ligation was performed by T4 DNA ligase and ligation mix [Triton X-100 (1%), 1 x Ligation buffer 10 X Ligation buffer- Tris-Cl pH 7.5 (500 mM), MgCl2 (100 mM), DTT (100 mM), BSA (0.105 mg/ml), ATP (1.05 mM)]. The ligated samples were purified by PCI and subjected to ethanol precipitation. The pellet was eluted in 1 X TE (pH 8.0) to obtain the 3 C library. The 4 C digestion was performed by NlaIII (50 units, NEB), and the samples were ligated, purified, and precipitated similar to the 3 C library to obtain the 4 C library. The 4 C library was subjected to RNaseA treatment and purified with the QIAquick PCR purification kit. The concentration of the library was then measured by Nanodrop and subjected to PCRs using the oligos for the enhancer viewpoint. The samples were next PCR purified using the same kit and subjected to next-generation sequencing with Illumina HiSeq2500/NOVA seq. The 4 C oligos are listed in Supplementary file 1e.

### 4C data analysis

The sequenced reads in FastQ file were demultiplexed by matching the appropriate primer sequences for each condition without allowing for any mismatches. Demultiplexed reads were processed using 4cseqpipe software. Restriction site tracks were created for the hg38 human genome by mentioning the restriction sites of the first cutter as GATC and that of the second cutter as CATG. The Phred scores of demultiplexed reads were changed to Phred64 format and the FastQ files were converted into raw format. Furthermore, valid 4 C reads were mapped to the generated restriction site tracks. Unique fragment ends/non-unique fragment ends were used. The mapped reads were normalized and near-cis domainograms at a maximum height of 0.1 were created by using the truncated log mean statistic with a trend resolution of 1 kb for the genomic region chr11:42300000–42400000.

### RNA isolation, cDNA synthesis, and PCR

Cells were lysed in 1 ml of TRIzol (Thermo Fisher Inc). 200 μl chloroform was added to the sample, briefly vortexed, and centrifuged at 12 K rpm for 12 min. The aqueous phase was carefully collected and transferred to the fresh tube. One volume of isopropanol was added to the sample and incubated at room temperature for 10 mins to precipitate the RNA. The samples were centrifuged at 12 K rpm for 12 min, supernatant was discarded without disturbing the pellet. The pellet obtained was washed with 75% ethanol. The pellet was air dried and dissolved in RNase-free water. RNA obtained was treated with ezDNase (Invitrogen) to remove the traces of contaminating DNA. 1 μg of RNA was used for each cDNA synthesis reaction by Superscript IV (Invitrogen) and random hexamers as per the manufacturer’s recommendation. The CFX96 touch (Bio-Rad) real-time PCR was used for qRT-PCRs. The fold changes were calculated by the ΔΔCt method and individual expression data was normalized to GAPDH mRNA. The *p*-values were calculated by Student’s unpaired two-tailed t-test from independent four biological replicates. qRT-PCR primers are listed in Supplementary file 1f.

### GRO-seq analysis

FastQ files from GEO accession number GSE43836 were downloaded from European Nucleotide Archive. Reads with base quality <20 in a sliding window of 4 bases and with a length of <36 were removed using Trimmomatic 0.39. Trimmed reads were aligned using bowtie2 2.5.1 with default parameters. Duplicate reads from the alignment files were removed using samtools 1.16.1. De-duplicated aligned reads were assigned in a strand-specific manner to transcript feature of the hg38.ncbiRefSeq.gtf.gz file by allowing multi-mapping reads and considering the largest overlap in case of overlapping features using featureCounts v2.0.3. CPM normalised strand-specific signal files with a bin size of 1 bp were generated using bamCoverage 3.5.1. Differential gene expression analysis was performed with default parameters using DESeq2 1.36.0. Upregulated genes were defined as those with adjusted *p*-value <0.05 and log2(FC)>1 in their respective conditions. Top, middle, and bottom 10% of upregulated genes based on their base mean value in E2-40m vs VEH condition were subsetted as highly expressing, moderately expressing, and low expressing upregulated genes. The closest upregulated genes in E2-160m vs E2-40m near highly expressing, moderately expressing and low expressing upregulated genes in E2-40m vs VEH condition were identified using bedtools closest v2.30.0. Only genes within a genomic distance of 1 Mb were considered. Log2-transformed DEseq2 normalised counts with the addition of the arbitrary value 1 was used to compare the gene expression trends across time points and categories of upregulated genes. All plots were generated using R 4.2.2.

## Data Availability

All source data associated with the manuscript are provided as source data. Probe and primer sequences are provided as Supplementary file 1. The following previously published dataset was used: HahN MurakamiS NagariA DankoCG 2013Enhancer transcripts mark active estrogen receptor binding sitesNCBI Gene Expression OmnibusGSE4383610.1101/gr.152306.112PMC373009623636943

## Additional files

Supplementary file 1. Oligos and probe sequences. (a) InTFF3 probe sequence. (b) ExTFF3 probe sequence. (c) InTFF1 probe sequence. (d) ExTFF1 probe sequence. (e) 4C Seq oligo sequences. (f) qPCR oligo sequences

MDAR checklist

## References

[bib1] Arnesen S, Blanchard Z, Williams MM, Berrett KC, Li Z, Oesterreich S, Richer JK, Gertz J (2021). Estrogen receptor alpha mutations in breast cancer cells cause gene expression changes through constant activity and secondary effects. Cancer Research.

[bib2] Boehning M, Dugast-Darzacq C, Rankovic M, Hansen AS, Yu T, Marie-Nelly H, McSwiggen DT, Kokic G, Dailey GM, Cramer P, Darzacq X, Zweckstetter M (2018). RNA polymerase II clustering through carboxy-terminal domain phase separation. Nature Structural & Molecular Biology.

[bib3] Boija A, Klein IA, Sabari BR, Dall’Agnese A, Coffey EL, Zamudio AV, Li CH, Shrinivas K, Manteiga JC, Hannett NM, Abraham BJ, Afeyan LK, Guo YE, Rimel JK, Fant CB, Schuijers J, Lee TI, Taatjes DJ, Young RA (2018). Transcription factors activate genes through the phase-separation capacity of their activation domains. Cell.

[bib4] Bojcsuk D, Nagy G, Balint BL (2017). Inducible super-enhancers are organized based on canonical signal-specific transcription factor binding elements. Nucleic Acids Research.

[bib5] Buecker C, Srinivasan R, Wu Z, Calo E, Acampora D, Faial T, Simeone A, Tan M, Swigut T, Wysocka J (2014). Reorganization of enhancer patterns in transition from naive to primed pluripotency. Cell Stem Cell.

[bib6] Bulger M, Groudine M (1999). Looping versus linking: toward a model for long-distance gene activation. Genes & Development.

[bib7] Cai D, Feliciano D, Dong P, Flores E, Gruebele M, Porat-Shliom N, Sukenik S, Liu Z, Lippincott-Schwartz J (2019). Phase separation of YAP reorganizes genome topology for long-term YAP target gene expression. Nature Cell Biology.

[bib8] Chen H, Levo M, Barinov L, Fujioka M, Jaynes JB, Gregor T (2018). Dynamic interplay between enhancer-promoter topology and gene activity. Nature Genetics.

[bib9] Chen L, Zhang Z, Han Q, Maity BK, Rodrigues L, Zboril E, Adhikari R, Ko SH, Li X, Yoshida SR, Xue P, Smith E, Xu K, Wang Q, Huang THM, Chong S, Liu Z (2023). Hormone-induced enhancer assembly requires an optimal level of hormone receptor multivalent interactions. Molecular Cell.

[bib10] Chepelev I, Wei G, Wangsa D, Tang Q, Zhao K (2012). Characterization of genome-wide enhancer-promoter interactions reveals co-expression of interacting genes and modes of higher order chromatin organization. Cell Research.

[bib11] Chinery R, Williamson J, Poulsom R (1996). The gene encoding human intestinal trefoil factor (TFF3) is located on chromosome 21q22.3 clustered with other members of the trefoil peptide family. Genomics.

[bib12] Cho WK, Spille JH, Hecht M, Lee C, Li C, Grube V, Cisse II (2018). Mediator and RNA polymerase II clusters associate in transcription-dependent condensates. Science.

[bib13] Chong S, Dugast-Darzacq C, Liu Z, Dong P, Dailey GM, Cattoglio C, Heckert A, Banala S, Lavis L, Darzacq X, Tjian R (2018). Imaging dynamic and selective low-complexity domain interactions that control gene transcription. Science.

[bib14] Chong S, Graham TGW, Dugast-Darzacq C, Dailey GM, Darzacq X, Tjian R (2022). Tuning levels of low-complexity domain interactions to modulate endogenous oncogenic transcription. Molecular Cell.

[bib15] Clark MB, Mercer TR, Bussotti G, Leonardi T, Haynes KR, Crawford J, Brunck ME, Cao KAL, Thomas GP, Chen WY, Taft RJ, Nielsen LK, Enright AJ, Mattick JS, Dinger ME (2015). Quantitative gene profiling of long noncoding RNAs with targeted RNA sequencing. Nature Methods.

[bib16] Conesa A, Madrigal P, Tarazona S, Gomez-Cabrero D, Cervera A, McPherson A, Szcześniak MW, Gaffney DJ, Elo LL, Zhang X, Mortazavi A (2016). A survey of best practices for RNA-seq data analysis. Genome Biology.

[bib17] Coté A, O’Farrell A, Dardani I, Dunagin M, Coté C, Wan Y, Bayatpour S, Drexler HL, Alexander KA, Chen F, Wassie AT, Patel R, Pham K, Boyden ES, Berger S, Phillips-Cremins J, Churchman LS, Raj A (2023). Post-transcriptional splicing can occur in a slow-moving zone around the gene. eLife.

[bib18] Coulon A, Ferguson ML, de Turris V, Palangat M, Chow CC, Larson DR (2014). Kinetic competition during the transcription cycle results in stochastic RNA processing. eLife.

[bib19] Dixon BS, Sharma RV, Dennis MJ (1996). The bradykinin B2 receptor is a delayed early response gene for platelet-derived growth factor in arterial smooth muscle cells. The Journal of Biological Chemistry.

[bib20] Drexler HL, Choquet K, Churchman LS (2020). Splicing kinetics and coordination revealed by direct nascent RNA sequencing through nanopores. Molecular Cell.

[bib21] Femino AM, Fay FS, Fogarty K, Singer RH (1998). Visualization of single RNA transcripts in situ. Science.

[bib22] Fowler T, Sen R, Roy AL (2011). Regulation of primary response genes. Molecular Cell.

[bib23] Freter RR, Alberta JA, Hwang GY, Wrentmore AL, Stiles CD (1996). Platelet-derived growth factor induction of the immediate-early gene MCP-1 is mediated by NF-kappaB and a 90-kDa phosphoprotein coactivator. The Journal of Biological Chemistry.

[bib24] Friedman MJ, Wagner T, Lee H, Rosenfeld MG, Oh S (2024). Enhancer–promoter specificity in gene transcription: molecular mechanisms and disease associations. Experimental & Molecular Medicine.

[bib25] Furlong EEM, Levine M (2018). Developmental enhancers and chromosome topology. Science.

[bib26] Galouzis CC, Furlong EEM (2022). Regulating specificity in enhancer-promoter communication. Current Opinion in Cell Biology.

[bib27] Gamliel A, Meluzzi D, Oh S, Jiang N, Destici E, Rosenfeld MG, Nair SJ (2022). Long-distance association of topological boundaries through nuclear condensates. PNAS.

[bib28] Hah N, Danko CG, Core L, Waterfall JJ, Siepel A, Lis JT, Kraus WL (2011). A rapid, extensive, and transient transcriptional response to estrogen signaling in breast cancer cells. Cell.

[bib29] Hah N, Murakami S, Nagari A, Danko CG, Kraus WL (2013). Enhancer transcripts mark active estrogen receptor binding sites. Genome Research.

[bib30] Haimovich G, Gerst JE (2018). Single-molecule Fluorescence *in situ* Hybridization (smFISH) for RNA detection in adherent animal cells. Bio-Protocol.

[bib31] Herschman HR (1991). Primary response genes induced by growth factors and tumor promoters. Annual Review of Biochemistry.

[bib32] Khodor YL, Menet JS, Tolan M, Rosbash M (2012). Cotranscriptional splicing efficiency differs dramatically between Drosophila and mouse. RNA.

[bib33] Koşar Z, Erbaş A (2022). Can the concentration of a transcription factor affect gene expression?. Frontiers in Soft Matter.

[bib34] Kwon S (2013). Single-molecule fluorescence in situ hybridization: quantitative imaging of single RNA molecules. BMB Reports.

[bib35] Lebedeva G, Yamaguchi A, Langdon SP, Macleod K, Harrison DJ (2012). A model of estrogen-related gene expression reveals non-linear effects in transcriptional response to tamoxifen. BMC Systems Biology.

[bib36] Levenson AS, Jordan VC (1997). MCF-7: the first hormone-responsive breast cancer cell line. Cancer Research.

[bib37] Levine M, Tjian R (2003). Transcription regulation and animal diversity. Nature.

[bib38] Li W, Notani D, Ma Q, Tanasa B, Nunez E, Chen AY, Merkurjev D, Zhang J, Ohgi K, Song X, Oh S, Kim HS, Glass CK, Rosenfeld MG (2013). Functional roles of enhancer RNAs for oestrogen-dependent transcriptional activation. Nature.

[bib39] Liu Z, Merkurjev D, Yang F, Li W, Oh S, Friedman MJ, Song X, Zhang F, Ma Q, Ohgi KA, Krones A, Rosenfeld MG (2014). Enhancer activation requires trans-recruitment of a mega transcription factor complex. Cell.

[bib40] Mann R, Notani D (2023). Transcription factor condensates and signaling driven transcription. Nucleus.

[bib41] Masiakowski P, Breathnach R, Bloch J, Gannon F, Krust A, Chambon P (1982). Cloning of cDNA sequences of hormone-regulated genes from the MCF-7 human breast cancer cell line. Nucleic Acids Research.

[bib42] Nair SJ, Yang L, Meluzzi D, Oh S, Yang F, Friedman MJ, Wang S, Suter T, Alshareedah I, Gamliel A, Ma Q, Zhang J, Hu Y, Tan Y, Ohgi KA, Jayani RS, Banerjee PR, Aggarwal AK, Rosenfeld MG (2019). Phase separation of ligand-activated enhancers licenses cooperative chromosomal enhancer assembly. Nature Structural & Molecular Biology.

[bib43] Oh S, Shao J, Mitra J, Xiong F, D’Antonio M, Wang R, Garcia-Bassets I, Ma Q, Zhu X, Lee J-H, Nair SJ, Yang F, Ohgi K, Frazer KA, Zhang ZD, Li W, Rosenfeld MG (2021). Enhancer release and retargeting activates disease-susceptibility genes. Nature.

[bib44] Panigrahi A, O’Malley BW (2021). Mechanisms of enhancer action: the known and the unknown. Genome Biology.

[bib45] Patange S, Ball DA, Wan Y, Karpova TS, Girvan M, Levens D, Larson DR (2022). MYC amplifies gene expression through global changes in transcription factor dynamics. Cell Reports.

[bib46] Ptashne M (1986). Gene regulation by proteins acting nearby and at a distance. Nature.

[bib47] Quintin J, Le Péron C, Palierne G, Bizot M, Cunha S, Sérandour AA, Avner S, Henry C, Percevault F, Belaud-Rotureau MA, Huet S, Watrin E, Eeckhoute J, Legagneux V, Salbert G, Métivier R (2014). Dynamic estrogen receptor interactomes control estrogen-responsive trefoil Factor (TFF) locus cell-specific activities. Molecular and Cellular Biology.

[bib48] Raj A, Peskin CS, Tranchina D, Vargas DY, Tyagi S (2006). Stochastic mRNA synthesis in mammalian cells. PLOS Biology.

[bib49] Raj A, van den Bogaard P, Rifkin SA, van Oudenaarden A, Tyagi S (2008). Imaging individual mRNA molecules using multiple singly labeled probes. Nature Methods.

[bib50] Rao SSP, Huntley MH, Durand NC, Stamenova EK, Bochkov ID, Robinson JT, Sanborn AL, Machol I, Omer AD, Lander ES, Aiden EL (2014). A 3D map of the human genome at kilobase resolution reveals principles of chromatin looping. Cell.

[bib51] Rodriguez J, Ren G, Day CR, Zhao K, Chow CC, Larson DR (2019). Intrinsic dynamics of a human gene reveal the basis of expression heterogeneity. Cell.

[bib52] Ryu K, Park G, Cho WK (2024). Emerging insights into transcriptional condensates. Experimental & Molecular Medicine.

[bib53] Sabari BR, Dall’Agnese A, Boija A, Klein IA, Coffey EL, Shrinivas K, Abraham BJ, Hannett NM, Zamudio AV, Manteiga JC, Li CH, Guo YE, Day DS, Schuijers J, Vasile E, Malik S, Hnisz D, Lee TI, Cisse II, Roeder RG, Sharp PA, Chakraborty AK, Young RA (2018). Coactivator condensation at super-enhancers links phase separation and gene control. Science.

[bib54] Sanyal A, Lajoie BR, Jain G, Dekker J (2012). The long-range interaction landscape of gene promoters. Nature.

[bib55] Saravanan B, Soota D, Islam Z, Majumdar S, Mann R, Meel S, Farooq U, Walavalkar K, Gayen S, Singh AK, Hannenhalli S, Notani D (2020). Ligand dependent gene regulation by transient ERα clustered enhancers. PLOS Genetics.

[bib56] Sha Y, Phan JH, Wang MD (2015). Effect of low-expression gene filtering on detection of differentially expressed genes in RNA-seq data.

[bib57] Shrinivas K, Sabari BR, Coffey EL, Klein IA, Boija A, Zamudio AV, Schuijers J, Hannett NM, Sharp PA, Young RA, Chakraborty AK (2019). Enhancer features that drive formation of transcriptional condensates. Molecular Cell.

[bib58] Skinner SO, Xu H, Nagarkar-Jaiswal S, Freire PR, Zwaka TP, Golding I (2016). Single-cell analysis of transcription kinetics across the cell cycle. eLife.

[bib59] Stortz M, Pecci A, Presman DM, Levi V (2020). Unraveling the molecular interactions involved in phase separation of glucocorticoid receptor. BMC Biology.

[bib60] Stortz M, Presman DM, Levi V (2024). Transcriptional condensates: a blessing or a curse for gene regulation?. Communications Biology.

[bib61] Stossi F, Dandekar RD, Mancini MG, Gu G, Fuqua SAW, Nardone A, De Angelis C, Fu X, Schiff R, Bedford MT, Xu W, Johansson HE, Stephan CC, Mancini MA (2020). Estrogen-induced transcription at individual alleles is independent of receptor level and active conformation but can be modulated by coactivators activity. Nucleic Acids Research.

[bib62] Svensson V, Natarajan KN, Ly LH, Miragaia RJ, Labalette C, Macaulay IC, Cvejic A, Teichmann SA (2017). Power analysis of single-cell RNA-sequencing experiments. Nature Methods.

[bib63] Tarazona S, García-Alcalde F, Dopazo J, Ferrer A, Conesa A (2011). Differential expression in RNA-seq: a matter of depth. Genome Research.

[bib64] Uyehara CM, Apostolou E (2023). 3D enhancer-promoter interactions and multi-connected hubs: Organizational principles and functional roles. Cell Reports.

[bib65] Vandenberg LN, Colborn T, Hayes TB, Heindel JJ, Jacobs DR, Lee DH, Shioda T, Soto AM, vom Saal FS, Welshons WV, Zoeller RT, Myers JP (2012). Hormones and endocrine-disrupting chemicals: low-dose effects and nonmonotonic dose responses. Endocrine Reviews.

[bib66] van de Werken HJG, de Vree PJP, Splinter E, Holwerda SJB, Klous P, de Wit E, de Laat W (2012). 4C technology: protocols and data analysis. Methods in Enzymology.

[bib67] Williams M, Lyu MS, Yang YL, Lin EP, Dunbrack R, Birren B, Cunningham J, Hunter K (1999). Ier5, a novel member of the slow-kinetics immediate-early genes. Genomics.

[bib68] Winkles JA, Moldave K (1997). Progress in Nucleic Acid Research and Molecular Biology.

[bib69] Winston JT, Pledger WJ (1993). Growth factor regulation of cyclin D1 mRNA expression through protein synthesis-dependent and -independent mechanisms. Molecular Biology of the Cell.

[bib70] Yamamoto KR, Alberts BM (1976). Steroid receptors: elements for modulation of eukaryotic transcription. Annual Review of Biochemistry.

[bib71] Yan J, Chen SAA, Local A, Liu T, Qiu Y, Dorighi KM, Preissl S, Rivera CM, Wang C, Ye Z, Ge K, Hu M, Wysocka J, Ren B (2018). Histone H3 lysine 4 monomethylation modulates long-range chromatin interactions at enhancers. Cell Research.

[bib72] Zabidi MA, Arnold CD, Schernhuber K, Pagani M, Rath M, Frank O, Stark A (2015). Enhancer-core-promoter specificity separates developmental and housekeeping gene regulation. Nature.

[bib73] Zambrano S, Loffreda A, Carelli E, Stefanelli G, Colombo F, Bertrand E, Tacchetti C, Agresti A, Bianchi ME, Molina N, Mazza D (2020). First responders shape a prompt and sharp NF-κB-mediated transcriptional response to TNF-α. iScience.

[bib74] Zenklusen D, Larson DR, Singer RH (2008). Single-RNA counting reveals alternative modes of gene expression in yeast. Nature Structural & Molecular Biology.

