# Supplementary material for: Ligand-dependent enhancer activation indirectly modulates non-target promoters in a chromatin domain
Source: eLife. 2026 Jul 7;13:RP102417. doi: 10.7554/eLife.102417 (PMC13341115; doi:10.7554/eLife.102417)
Supplement: Peer review history

# Editor report: eLife Assessment

**DOI:** 10.7554/eLife.102417.4.sa0

**Authors:** Melike Lakadamyali

**Affiliations:** University of Pennsylvania United States

**Keywords:** Important, Solid

The authors use single molecule imaging and in vivo loop-capture genomic approaches to investigate estrogen mediated enhancer-target gene activation in human cancer cells. Their results, which are supported by **solid** evidence and will be **important** for the field, suggest that ER-alpha can, in a temporal delay, activate a non-target gene TFF3, which is in proximity to the main target gene TFF1, through an indirect mechanism as the estrogen responsive enhancer does not loop with the TFF3 promoter. The mechanism of activation may involve condensate formation, however, more future work is needed to fully support a condensate based model. This work will be of interest to those studying transcriptional gene regulation and hormone-aggravated cancers.

# Referee report: Reviewer #1 (Public review)

**DOI:** 10.7554/eLife.102417.4.sa1

[Editors' note: this version has been assessed by the Reviewing Editor without further input from the original reviewers. The authors have addressed the comments raised in the previous round of review.]

Summary:

The manuscript by Bohra et al. describes the indirect effects of ligand-dependent gene activation on neighboring non-target genes. The authors utilized single-molecule RNA-FISH (targeting both mature and intronic regions), 4C-seq, and enhancer deletions to demonstrate that the non-enhancer-targeted gene TFF3, located in the same TAD as the target gene TFF1, alters its expression when TFF1 expression declines at the end of the estrogen signaling peak. Since the enhancer does not loop with TFF3, the authors conclude that mechanisms other than estrogen receptor or enhancer-driven induction are responsible for TFF3 expression. Moreover, ERα intensity correlations show that both high and low levels of ERα are unfavorable for TFF1 expression. The ERa level correlations are further supported by overexpression of GFP-ERa. The authors conclude that transcriptional machinery used by TFF1 for its acute activation can negatively impact the TFF3 at peak of signaling but once, the condensate dissolves, TFF3 benefits from it for its low expression.

Strengths:

The findings are indeed intriguing. The authors have maintained appropriate experimental controls, and their conclusions are well-supported by the data.

# Referee report: Reviewer #3 (Public review)

**DOI:** 10.7554/eLife.102417.4.sa2

Summary:

In this manuscript Bohra et al. measure the effects of estrogen responsive gene expression upon induction on nearby target genes using a TAD containing the genes TFF1 and TFF3 as a model. The authors propose that there is a sort competition for transcriptional machinery between TFF1 (estrogen responsive) and TFF3 (not responsive) such that when TFF1 is activated and machinery is recruited, TFF3 is activated after a time delay. The authors attribute this time delay to transcriptional machinery that was being sequestered at TFF1 becomes available to the proximal TFF3 locus. The authors demonstrate that this activation is not dependent on contact with the TFF1 enhancer through deletion, instead they conclude that it is dependent on a phase-separated condensate which can sequester transcriptional machinery. Although the manuscript reports an interesting observation that there is a dose dependence and time delay on the expression of TFF1 relative to TFF3, there is much room for improvement in the analysis and reporting of the data. Most importantly there is no direct test of condensate formation at the locus in the context of this study: i.e. dissolution upon the enhancer deletion, decay in a temporal manner, and dependence of TFF1 expression on condensate formation. Using 1,6' hexanediol to draw conclusion on this matter is not adequate to draw conclusions on the effect of condensates on a specific genes activity given current knowledge on its non-specificity and multitude of indirect effects. Thus, in my opinion the major claim that this effect of a time delayed expression of TFF3 being dependent on condensates in not supported by the current data.

Strengths:

The depends of TFF1 expression on a single enhancer and the temporal delay in TFF3 is a very interesting finding.

The non-linear dependence of TFF1 and TTF3 expression on ER concentration is very interesting with potentially broader implications.

The combined use of smFISH, enhancer deletion, and 4C to build a coherent model is a good approach.

# Author comment: Author response

**DOI:** 10.7554/eLife.102417.4.sa3

**Authors:** Darshika Bohra, Zubairul Islam, Sunderraj Nidharshan, Aprotim Mazumder, Dimple Notani

**Affiliations:** Tata Institute of Fundamental Research Hyderabad India; National Centre for Biological Sciences Bangalore India; School of Biotechnology, Amrita Vishwa Vidyapeetham, Kollam - 690525, Kerala Kollam India; TIFR Centre for Interdisciplinary Sciences Hyderabad India; National Centre for Biological Sciences Bangalore India

The following is the authors’ response to the previous reviews

We are pleased that Reviewer 3 appreciated our findings and found the temporal lag between the expression of TFF1 and TFF3 during signaling particularly interesting. The reviewer also advised us not to overemphasize that this lag arises from phase separation of ERα at the TFF1 locus, as the use of 1,6-hexanediol alone is not sufficient to conclusively establish whether ERα condensates undergo liquid–liquid phase separation.

We agree with this assessment and have revised the manuscript accordingly. Specifically, we have modified the title to remove reference to phase separation and have updated the text throughout the manuscript to avoid claiming that the observed condensates are a result of phase separation.

The revised title is:

**“**Ligand-dependent Enhancer Activation Indirectly Modulates Non-target Promoters in a Chromatin Domain.”

With these changes, we are proceeding with the version of record using revised version of the manuscript.

Thank you for your continued support.
